# Expression profiles of small non-coding RNAs in breast cancer tumors characterize clinicopathological features and show prognostic and predictive potential

**DOI:** 10.1038/s41598-022-26954-w

**Published:** 2022-12-30

**Authors:** Emmi Kärkkäinen, Sami Heikkinen, Maria Tengström, Veli-Matti Kosma, Arto Mannermaa, Jaana M. Hartikainen

**Affiliations:** 1 School of Medicine, Institute of Clinical Medicine, Pathology and Forensic Medicine, and Translational Cancer Research Area, University of Eastern Finland, Yliopistonranta 1 C, 70210 Kuopio, Finland; 2 School of Medicine, Institute of Clinical Medicine, University of Eastern Finland, Kuopio, Finland; 3 School of Medicine, Institute of Biomedicine, University of Eastern Finland, Kuopio, Finland; 4 School of Medicine, Institute of Clinical Medicine, Oncology, and Cancer Center of Eastern Finland, University of Eastern Finland, Kuopio, Finland; 5 Cancer Center, Kuopio University Hospital, Kuopio, Finland; 6 Department of Clinical Pathology, Kuopio University Hospital, Kuopio, Finland

**Keywords:** Breast cancer, Diagnostic markers, Predictive markers, Prognostic markers, Cancer, Small RNAs

## Abstract

Precision medicine approaches are required for more effective therapies for cancer. As small non-coding RNAs (sncRNAs) have recently been suggested as intriguing candidates for cancer biomarkers and have shown potential also as novel therapeutic targets, we aimed at profiling the non-miRNA sncRNAs in a large sample set to evaluate their role in invasive breast cancer (BC). We used small RNA sequencing and 195 fresh-frozen invasive BC and 22 benign breast tissue samples to identify significant associations of small nucleolar RNAs, small nuclear RNAs, and miscellaneous RNAs with the clinicopathological features and patient outcome of BC. Ninety-six and five sncRNAs significantly distinguished (*Padj* < 0.01) invasive local BC from benign breast tissue and metastasized BC from invasive local BC, respectively. Furthermore, 69 sncRNAs significantly associated (*Padj* < 0.01) with the tumor grade, hormone receptor status, subtype, and/or tumor histology. Additionally, 42 sncRNAs were observed as candidates for prognostic markers and 29 for predictive markers for radiotherapy and/or tamoxifen response (*P* < 0.05). We discovered the clinical relevance of sncRNAs from each studied RNA type. By introducing new sncRNA biomarker candidates for invasive BC and validating the potential of previously described ones, we have guided the way for further research that is warranted for providing novel insights into BC biology.

## Introduction

Women’s breast cancer (BC) is currently the most frequently occurring cancer^1^. The heterogeneity of the disease results in different outcomes even in cases that share similar prognostic and/or predictive clinicopathological or molecular features that are commonly used for BC classification. The search for novel biomarkers that would distinguish BC patients at diagnosis is continuous and the small noncoding RNAs (sncRNAs) represent an alternative option for identifying prognostic and predictive markers for BC. They show promise as biomarkers as they can be easily detected from the serum and plasma of patients, thus offering a non-invasive method for diagnosis, prognosis, and evaluation of treatment efficacy^2,3^. However, more information on their expression in tumors, and thus suitability as biomarkers is still required.

The group of small noncoding RNAs (sncRNAs) consists of different regulatory RNAs including microRNAs (miRNAs), Piwi-interacting RNAs (piRNAs), small nucleolar RNAs (snoRNAs), small nuclear RNAs (snRNAs), and miscellaneous RNAs (miscRNAs). They have essential roles in maintaining vital cellular functions through various mechanisms usually including associations with specific proteins and the formation of RNA–protein complexes^4–6^. Here we have concentrated on the non-miRNAs/piRNAs as miRNAs’ contributions to pathogenicity and tumorigenesis have been extensively studied and piRNAs were discussed in detail in our recent study^7^.

The majority of snoRNAs are processed from the introns of non-coding or protein-coding genes, locate predominantly in the nucleolus, and are required for the cleavage of pre-ribosomal RNAs (pre-rRNAs) as well as for the processing of rRNAs and other RNA types. snoRNAs can be classified into two major groups based on their structure: C/D box snoRNAs and H/ACA box snoRNAs^8^. Furthermore, small Cajal body-specific RNAs (scaRNAs), a minor subgroup of snoRNAs, locate specifically in the nuclear Cajal bodies and possess both C/D and H/ACA boxes^9^. Some snoRNAs, called orphan snoRNAs, do not appear to target identified sncRNAs and have been suggested to engage in alternative splicing and implied to have tissue-specific roles^10–12^. Further demonstrating their versatility, the ability of snoRNAs to act as precursors to shorter miRNA-like snoRNA-derived RNAs (sdRNAs) was recently observed^13^. C/D box and H/ACA box snoRNAs associate with various proteins to form ribonucleoproteins (snoRNPs)^8,9^. Also the predominantly nucleus-localized snRNAs exist in two types with different sequence features and interacting proteins^8^. snRNAs are crucial for RNA splicing and have also been observed to function in transcription regulation and inhibition of mRNA polyadenylation leading to mRNA decay^8,14^. miscRNAs in turn consist of a group of miscellaneous small RNAs including vault RNAs (VTRNAs), Y RNAs, and signal recognition particle (SRP) RNAs. Although the exact functions of VTRNAs remain scarce, they have been indicated to be involved in various cellular processes including intracellular transport, apoptosis, drug resistance, and autophagy either independently or in cooperation with vault proteins^15^. Y RNAs have been reported to act in RNA stability and cellular stress response and are fundamental for the initiation of DNA replication. SRP RNA in turn is part of SRP complex that plays an important role in translocating membrane and secretory proteins to the endoplasmic reticulum membrane^16^.

Since these sncRNAs are involved in diverse essential cellular processes, it is not surprising that they have been indicated to affect multiple human pathologies including cancer. The aberrant levels of many snoRNAs, miscRNAs, snRNAs, and sncRNA-derived fragments have been detected in various tumor types compared to normal/benign tissue^17–19^. Several sncRNAs and sncRNA-derived fragments have also been linked to cancer prognosis and drug resistance indicating their potential as prognostic and predictive markers for cancer^18,20–26^. Although the importance of sncRNAs in cellular homeostasis has been widely acknowledged, the mode of function of these RNAs in tumorigenesis remains widely unknown. Furthermore, a notable part of the thus far conducted sncRNA biomarker research has been performed using either small sample numbers and/or mainly PCR or microarrays instead of utilizing technologies that are more sensitive^23,24,27–30^. Since comprehensive evidence of the non-miRNA sncRNAs’ potential as prognostic and predictive markers in BC is still lacking, more research is required to investigate the associations of non-miRNA sncRNAs with clinical characteristics and patient outcome for providing information of their possible prognostic and predictive value.

Therefore, we have performed small RNA sequencing (RNA-seq) for 195 tumor samples from the Eastern Finnish Kuopio Breast Cancer Project (KBCP) to identify sncRNAs and their associations with the clinicopathological features of invasive local BC (ilBC) and patient outcome, and thus provide further evidence of the potential relevance of sncRNAs to BC in the pursue to better understand BC pathology.

## Materials and methods

### Sample material, RNA extraction, and small RNA-seq

The used sample material, RNA extraction, and small RNA-seq protocols have been described previously^7^. We used a set of 228 fresh-frozen tissue samples from 228 individuals from the KBCP material which has been previously described in more detail^7,31,32^. Altogether, we had BC tissue sample from 195 patients with invasive BC, and from 6 patients with in situ tumors, 22 representative breast tissue samples from 22 patients with a benign breast disease (no cancer), and a sample of normal breast tissue from 5 additional subjects without cancer or benign breast disease diagnosis. All tissue samples were collected during the diagnostic or treatment protocols in Kuopio University Hospital, and the BC tumor samples were collected from the BC patients (195 invasive and 6 in situ) in cancer surgery before other treatments. The clinicopathological, and treatment data relating to the tumors and patients used in this study is shown in Supplementary Table S1.

### Ethics approval and consent to participate

The study was conducted according to the guidelines of the Declaration of Helsinki, and approved by the joint Ethics Committee of the University of Eastern Finland and Kuopio University Hospital (Ethics Committee, Hospital District of Northern Savo) (approvals 7/89, 5.12.1989 and 225/2008, 21.10.2008). Informed signed consent was obtained from all subjects involved in the study.

### Bioinformatic data analysis

Small RNA-Seq preprocessing consisted of read quality assessment (FastQC, 0.10.1) and adapter trimming (TRIMMOMATIC, v0.39 with essential parameters: ILLUMINACLIP: TruSeqSmallRNA.fa:0:30:10, MINLEN 18, AVGQUAL:30)^33,34^. Reads aligning to mitochondrial DNA or rRNA, or composed of a single nucleotide, were removed using STAR (version 2.5.4b)^35^ with essential parameters as in ref except --outFilterMismatchNmax 2 and --outFilterMultimapNmax 100. The human sncRNA transcriptome was constructed by supplementing the short RNA genes (gene_types: snRNA, snoRNA, rRNA, Mt_tRNA, Mt_rRNA, misc_RNA, ribozyme, sRNA, scaRNA and vaultRNA) from the GENCODE v32 transcriptome with unique, additional short RNA genes (biotypes: misc_RNA, scRNA, snRNA, and snoRNA) from the “latest” NCBI hg38 transcriptome (accessed Feb 10, 2020). The final transcriptome consisted of 5331 RNAs out of which 532 overlapped an exon of a non-short RNA; these were included in alignment and gene-wise read counting but excluded from all statistical analyses (see below). Preprocessed reads were aligned to human genome version GRCh38 (primary assembly) using STAR (v2.5.4b), with essential alignment parameters as in ref.^36^ except --alignSJoverhangMin 1000, --alignSJDBoverhangMin 1, --alignIntronMin 20, --alignIntronMax 1000000, --outFilterMismatchNmax 5, --outFilterMismatchNoverReadLmax 0.1, and --outFilterType BySJout, to account for the presence of multiexonic sncRNAs. Gene-wise counts of primary alignments that overlapped a transcriptome member by at least 80% of the read length (but at least 15nt) were collected using the R (v3.6.1) function Rsubread::featureCounts (v2.0.1) with essential parameters: minOverlap = 15, fracOverlap = 0.80, largestOverlap = TRUE, minMQS = 1, primaryOnly = TRUE, strandSpecific = 1, and maxMOp = 1^37^. For uses other than differential gene expression (DEG) analysis, read counts were normalized using R function DESeq2::varianceStabilizingTransformation (vst) in “blind” mode (v1.26.0)^38^. To identify technical bias, quality control and exploration were performed (uniform manifold approximation and projection [UMAP], multidimensional scaling, principal component analysis, and unsupervised hierarchical clustering) in R/Bioconductor^39^; no bias assignable to e.g. library preparation batch or sequencing run was found.

### Statistical analyses

Statistically differentially expressed (DE) (*Padj* < 0.01) sncRNAs were identified using R (v3.6.1) package DESeq2 (v1.26.0), using Wald as test type, FDR for *P*-value adjustment, and the R function DESeq2::lfcShrink for shrinking fold changes of low expressed RNAs. The following clinical variables and BC subtypes were tested in the DEG analyses: ilBC versus benign breast tissue, metastasized BC versus ilBC, estrogen receptor (ER) negative versus ER positive BC, progesterone receptor (PR) negative versus PR positive BC, HER2 negative versus HER2 positive BC, node positive versus node negative, triple-negative BC (TNBC) versus luminal BC, HER2-type versus TNBC, HER2-type versus non-HER2-type, TNBC versus luminal A, TNBC versus luminal B, TNBC versus non-TNBC, luminal B versus A, non-luminal versus luminal, tumor grade versus benign, BC subtypes versus benign, tumor histology versus benign, malign versus benign, tumor stage versus benign and tumor size versus benign. Tumor grades, sizes, histology types, and stages were compared pairwise within each variable.

Only cases with invasive, local disease (i.e. excluding cases with in situ carcinoma or distant metastases at diagnosis) were included in the survival analyses for relapse-free survival (RFS), BC-specific survival (BCSS) and overall survival (OS). RFS was defined as the time between the date of BC diagnosis and the date of recurrence. BCSS was defined as the time between the date of BC diagnosis and the date of death due to BC, and OS as the time between the date of BC diagnosis and the date of death due to any cause. For the analysis of 220 sncRNAs with the highest mean expression and variation (SD), cases were divided into quartiles by the normalized read count, Q1 denoting the quartile with the lowest read count. All survival analyses were performed, for a given sncRNA, by comparing each other quartile against Q1. If a clear significant association of the highest or the lowest quartiles with patient outcome was observed in this comparison, survival analyses were correspondingly performed for these sncRNAs by comparing other quartiles against the highest quartile (Q4), the lowest quartile (Q1) against other quartiles, or the higher level against the lower level according to median. Univariate survival analyses were performed using the R (v3.6.2) function survival::coxph (v3.1-11) that implements the Cox’s proportional hazards model. Multivariate survival analyses providing the hazard ratios (HR) and confidence intervals (CI) for death (for BCSS and OS) or recurrence (for RFS) were performed using the Cox’s proportional hazards model in a forward stepwise manner implemented in R function MASS::stepAIC v7.3-51.5^40^. The additional data included in the multivariate survival analyses as covariates were, for clinical parameters, tumor grade, tumor histology, tumor size, nodal status, ER status, PR status, HER2 status, and age at diagnosis, and for treatment parameters, radiotherapy (RT) (yes/no), adjuvant chemotherapy (CT) (yes/no), and adjuvant endocrine therapy (ET) (yes/no). In addition to all cases with invasive local disease, the multivariate survival analyses were also performed separately for specific patient groups, defined by the received treatment as follows:

- “RT-treated cases” (cases with ilBC, has received RT): Adjuvant CT (y/n), adjuvant ET (y/n) and clinical data included in the analysis as covariates.
- “Tamoxifen-treated cases” (cases with invasive local, ER positive BC, has received adjuvant tamoxifen for more than 2 months, but no adjuvant CT): RT (y/n) and clinical data included in the analysis as covariates.
- “Surgery-only cases” (cases with ilBC, has not received adjuvant ET, CT, or RT): Clinical data included in the analysis as covariates.

Patient groups “RT only” (ilBC, has received RT, but no adjuvant CT or ET) and “adjuvant CT-treated” (ilBC, has received adjuvant CT, but no adjuvant ET) were too small (n ≤ 27) for reliable statistical analysis.

Survival rate plots for the univariate analysis were generated using basic R plotting functions (v3.6.2), and for the fitted multivariate survival probability using the R package survminer::ggadjustedcurves (v0.4.9) with “marginal” as the method.

### RT-qPCR

RT‐qPCR was performed to confirm the presence of SNORA80E (ENSG00000207475.1), SNORD103B (NCBI: 692235), SNORD59A (ENSG00000207031.1), and SNORD104 (ENSG00000199753.1) in a subset of the breast tissue samples (n = 28). The reverse transcription (RT) was performed according to the manufacturers’ instructions using 10 ng of total RNA, the TaqMan MicroRNA Reverse Transcription Kit (Thermo Fisher Scientific, Waltham, MA, USA) and specific RT primers obtained from the Custom TaqMan Small RNA Assays (Thermo Fisher Scientific) for SNORA80E, SNORD103B, SNORD59A, SNORD104, and the endogenous control RNU48. qPCR was performed in triplicate using the Custom TaqMan Small RNA Assays for SNORA80E, SNORD103B, SNORD59A, SNORD104, and RNU48 (Thermo Fisher Scientific), TaqMan Universal PCR Master Mix II no UNG (Thermo Fisher Scientific), and the LightCycler 96 Instrument (Roche Diagnostics, Mannheim, Germany) according to manufacturers’ instructions.

The relative expression for each small RNA was calculated from the Cq values using the ΔΔCq method with a run calibrator sample (in LightCycler 96 software). Pearson correlation was used for calculating the correlation between the relative expression in log2 scale (RT‐qPCR) and the vst normalized read counts in log2 scale (small RNA‐seq).

## Results

In total, the small RNA-seq yielded 552.5 million raw reads for the 228 sequenced samples, ranging from 1.1 to 6.6 million per sample. Preprocessing decreased the amount of reads to 451.9 million and the per-sample range to 0.84–4.2 million. A total of 391.0 million reads aligned to the human genome, with the per-sample range from 0.73 to 3.9 million. Finally, a total of 13.3 million reads counted to all 5331 sncRNAs, or 6.6 million to those 4799 short RNAs that did not overlap with an exon of a non-short RNA, of which 4.72, 1.67, and 0.068 million represented the main sncRNA classes (gene_types), snoRNA, miscRNA, and snRNA, respectively. Vst-normalized expression for those 1949 genes that had any reads in any sample and key clinical parameters for all 228 samples are available as Supplementary Table S2. Since the sequencing was performed as 40nt reads, the reads represent fragments of sncRNAs instead of the whole-length RNAs. The RNA-seq measurements for select top DE small RNAs, detailed in subsequent sections, were validated using comparison to independent RNA-seq data and/or by RT-qPCR. As shown in the violin plot in Supplementary Fig. S1A for 6 small RNAs, both the means and variances of expression in invasive BC tumors corresponded very well between the KBCP (n = 186) and the independent ILRS (n = 40) materials. Furthermore, the expression measured by RT-qPCR of four of the above genes, SNORA80E (ENSG00000207475.1), SNORD103B (NCBI: 692235), SNORD59A (ENSG00000207031.1), and SNORD104 (ENSG00000199753.1) correlated statistically significantly with the respective small RNA‐seq measurements (Supplementary Fig. S1B).

The preliminary view using the UMAP clustering of all 228 samples suggests that differentially expressed sncRNAs might be present for most clinical variables, but not all (Supplementary Fig. S2). For example, samples with the same clinical characteristic appear to group together for the main and more fine-grained sample subtype, tumor grade, and ER and PR status. However, such grouping appears to be absent in the case of nodal status.

### sncRNAs distinguish ilBC from benign breast tissue and from metastasized BC

**Figure 1:**
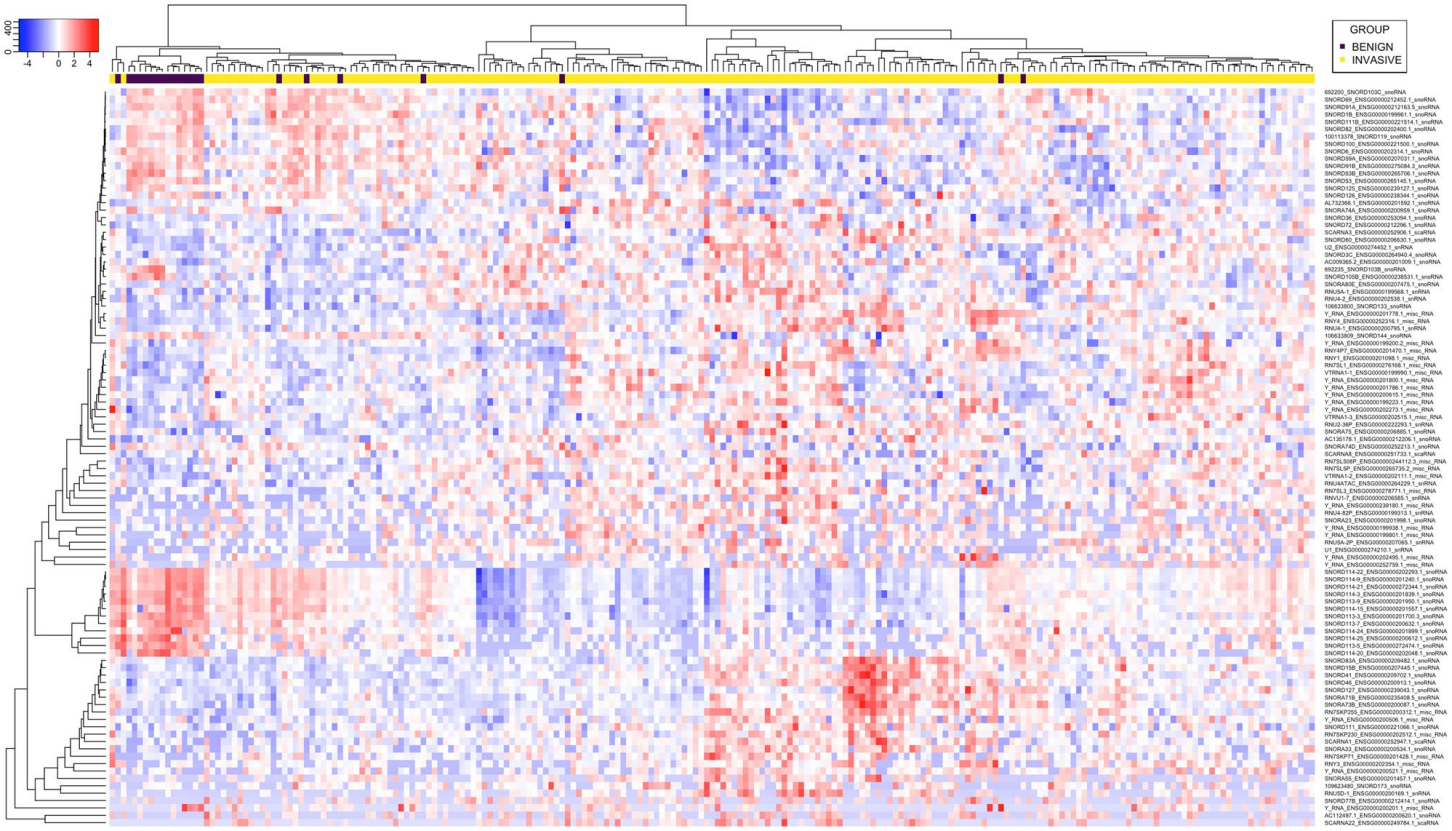
Ninety-six sncRNAs were found to significantly (*Padj* < 0.01) distinguish invasive local BC from benign breast tissue. The hierarchical clustering of 186 invasive local BC and 22 benign breast tissue samples (columns) and differentially expressed sncRNAs (rows) using Pearson metrics. Log2 fold change is marked by the color scale (from blue to red).

Altogether 63 sncRNAs were significantly (*Padj* < 0.01) upregulated and 33 sncRNAs downregulated in ilBC compared to benign breast tissue (Fig. 1, Supplementary Table S3).

A significant differential expression (*Padj* < 0.01) was also observed between metastasized BC and ilBC for five sncRNAs; two sncRNAs were upregulated and three sncRNAs downregulated in metastasized BC (M1 at diagnosis) when compared to ilBC (Supplementary Table S4).

### The hormone receptor status of the tumors associates with sncRNA expression

Altogether, 47 sncRNAs were significantly DE (*Padj* < 0.01) in the comparisons with the hormone receptor status of the tumors.

More specifically, 26 sncRNAs were upregulated in ER negative tumors (ilBC), whereas 18 sncRNAs were upregulated in ER positive tumors (ilBC) (Supplementary Table S5, Supplementary Fig. S3A).

Additionally, 11 sncRNAs were upregulated in PR negative tumors (ilBC) and three in PR positive tumors (Supplementary Table S6, Supplementary Fig. S3A). Except for two snoRNAs (SNORA2C and AL732366.1), all the sncRNAs that associated with the PR status of the tumors associated also with the ER status of the tumors (Supplementary Table S5, Supplementary Fig. S3A).

Only one sncRNA (SNORD124) associated with HER2 positive tumors in the HER2 negative versus HER2 positive tumors comparison (*P* = 2.77e−06, *Padj* = 2.95e−03, Log_2_FC =  − 1.020).

### Thirteen sncRNAs show prominent association with the tumor grade independently of the tumors’ ER status

**Figure 2:**
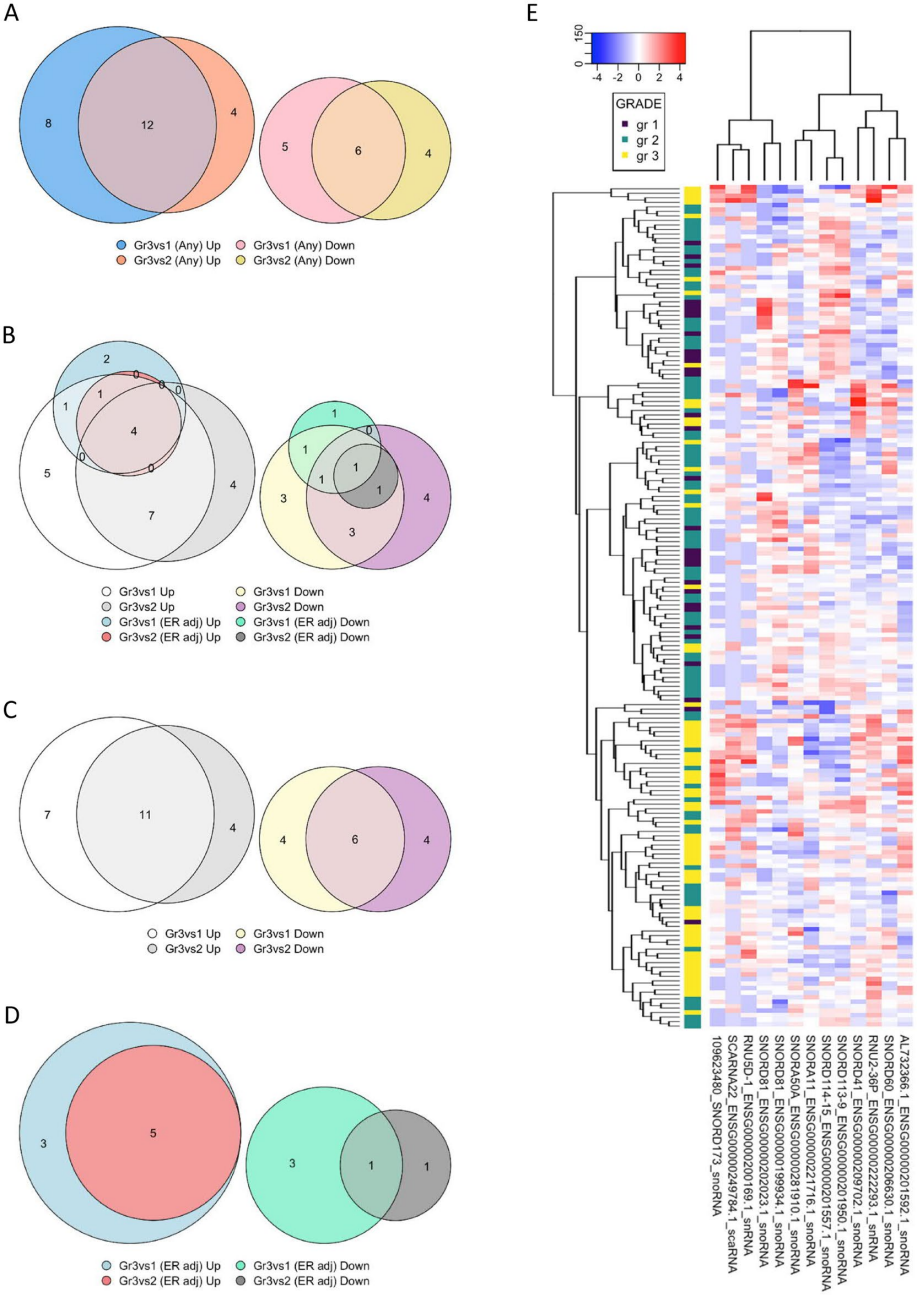
Thirty-nine sncRNAs in total associated (*Padj* < 0.01) with the tumor grade in ilBC cases; (**A**) and (**B**) twenty-four sncRNAs were upregulated, and 15 sncRNAs were downregulated in grade III tumors compared to grade I tumors and/or grade II tumors regardless of tumor ER status, (**C**) seventeen were shared in both gr. III versus gr. II, and gr. III versus gr. I when not adjusted for ER status, and (**D**) thirteen sncRNAs were significantly associated (*Padj* < 0.01) with the tumor grade independently of the ER status. (**E**) The hierarchical clustering of 186 invasive local BC samples (grade I n = 31, grade II n = 89, grade III n = 66) (columns) and differentially expressed sncRNAs (rows) using Euclidean metrics. Log2 fold change is marked by the color scale (from blue to red).

Thirty-nine sncRNAs in total associated (*Padj* < 0.01) with the tumor grade in ilBC cases (with or without adjusting for tumor ER status); 24 sncRNAs were upregulated and 15 sncRNAs were downregulated in grade III tumors compared to grade I tumors and/or grade II tumors (Supplementary Table S7, Fig. 2A,B). Of these 39 associations, 17 were shared in both comparisons (gr. III vs. gr. II, and gr. III vs. gr. I) when not adjusted for ER status (Fig. 2C). Thirteen sncRNAs associated with the tumor grade independently of the tumors’ ER status (Supplementary Table S7).

To summarize, the tumor grade-associated sncRNAs included five sncRNAs that were downregulated and eight sncRNAs that were upregulated in the advanced grade III tumors independently of the tumors’ ER status (Fig. 2D,E).

### SNORD99 and VTRNA1-1 associate with ductal tumor histology

SNORD99 and VTRNA1-1 showed association (*Padj* < 0.01) with ductal carcinoma in the comparison of lobular carcinoma versus ductal carcinoma (ilBC) (*P* = 1.72e − 04, *Padj* = 9.72e − 03, Log_2_FC =  − 0.561 and *P* = 1.50e − 05, *Padj* = 1.69e − 03, Log_2_FC =  − 0.745, respectively). The associations were observed also in the analysis adjusted by ER status, even though they reached the statistical significance only at the level of *Padj* < 0.05 (*P* = 3.51e − 04, *Padj* = 2.73e − 02, Log_2_FC =  − 0.520 and *P* = 7.12e − 04, *Padj* = 2.73e − 02, Log_2_FC =  − 0.552, respectively).

### A profile of sncRNAs define the tumors of luminal and TNBC subtype

**Figure 3:**
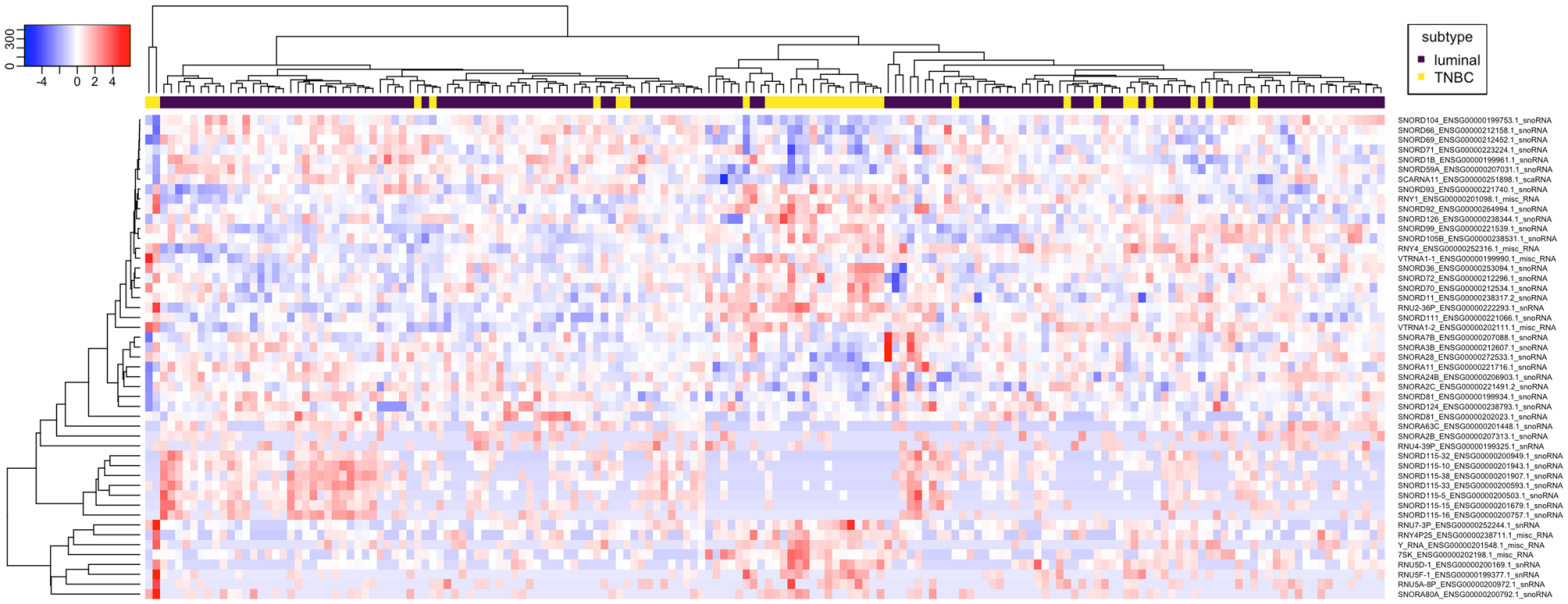
Forty-nine sncRNAs were found as characteristic (*Padj* < 0.01) to TNBC or luminal BC. The hierarchical clustering of 33 TNBC and 133 luminal BC samples (invasive local disease) (columns) and differentially expressed sncRNAs (rows) using Pearson metrics. Log2 fold change is marked by the color scale (from blue to red).

Altogether 23 sncRNAs were significantly (*Padj* < 0.01) upregulated in TNBC compared to luminal BC (ilBC), whereas 26 sncRNAs were significantly upregulated in luminal BC compared to TNBC (ilBC) (Fig. 3, Supplementary Table S8, Supplementary Fig. S3B).

Nine of the 23 TNBC-associated sncRNAs [SNORD111, SNORD92, SNORD72, RNU2-36P, RNU5D-1, RNU5F-1, RNY4, VTRNA1-1, and ENSG00000201548.1 (Y_RNA)] were also upregulated in ER negative and in PR negative tumors compared to ER positive and PR positive tumors, respectively (Table 1, Supplementary Figs. S3B, S4–S12). All but RNY4 significantly associated also with higher grade tumors, of which RNU2-36P and RNU5D-1 independently of the ER status (Supplementary Figs. S4–S9, S11, S12). Notably, the expression levels of SNORD111, RNU5D-1, RNU5F-1, and ENSG00000201548.1 were low (Supplementary Table S2).

**Table 1:** The sncRNAs that were significantly DE (*Padj* < 0.01) in the comparisons of hormone receptor status (ER negative vs. positive tumors and PR negative vs. positive tumors) and in the comparison between TNBC and luminal BC subtype indicating their potential role in invasive local BC. The significant associations (*Padj* < 0.01) for these sncRNAs with tumor grade are also shown.

Accession^a^	Name^b^	ER − versus ER +	PR − versus PR +	TNBC versus luminal BC	Gr III versus I	Gr III versus II
ENSG00000221066.1	SNORD111^c,e^	Up	Up	Up	Up	Up
ENSG00000264994.1	SNORD92	Up	Up	Up	Up	Up
ENSG00000212296.1	SNORD72^c^	Up	Up	Up	Up	
ENSG00000222293.1	RNU2-36P^c^	Up	Up	Up	Up*	Up*
ENSG00000200169.1	RNU5D-1^c,e^	Up	Up	Up	Up*	Up*
ENSG00000199377.1	RNU5F-1^e^	Up	Up	Up	Up	Up
ENSG00000252316.1	RNY4^c^	Up	Up	Up		
ENSG00000199990.1	VTRNA1-1^c^	Up	Up	Up	Up	Up
ENSG00000201548.1	Y_RNA^e^	Up	Up	Up		Up
ENSG00000221716.1	SNORA11	Down	Down	Down	Down	Down*
ENSG00000199753.1	SNORD104^d^	Down	Down	Down		Down

The asterisk denotes the significant association with tumor grade also independently of the ER status. More information can be found in the Supplementary material.

^a^Accession code from the ‘gene_id’ field from GENCODE.

^b^Name from the ‘gene_name’ field from GENCODE.

^c^Significantly upregulated also in invasive local BC compared to benign breast tissue.

^d^Significantly upregulated also in metastasized BC compared to invasive local BC.

^e^Note that the expression level of these RNAs was low.

*sncRNAs* small non-coding RNAs, *DE* differentially expressed, *Padj* adjusted *P*-value, *ER* estrogen receptor, *PR* progesterone receptor, *TNBC* triple-negative breast cancer, *Gr* grade.

Additionally, the luminal BC-associated SNORA11 and SNORD104 were also upregulated in ER positive and in PR positive tumors when compared to ER negative and PR negative tumors, respectively (Table 1, Supplementary Figs. S3B, S13 and S14). Both of them significantly associated also with lower grade but only SNORA11 independently of the ER status (Supplementary Figs. S13, S14).

### sncRNAs identified as candidate prognostic markers for BC; a group of them specifically for ER positive BC

Altogether 42 sncRNAs were identified as possible prognostic markers for ilBC. This group includes sncRNAs that associated with patient outcome (Overall *P* < 0.05 and Q4 *P* < 0.05) only in the analyses that were not restricted to any specific therapy groups, i.e. in analyses including all invasive cases, ER positive cases or ER negative cases separately, and/or in the cases who had received only surgery (all invasive cases who received only surgery, and ER positive cases with only surgery). Also, sncRNAs that associated with patient outcome only in the forementioned groups and in the cases who had received RT were included in these 42 sncRNAs (Supplementary Tables S9–S16, Supplementary Fig. S15A).

**Figure 4:**
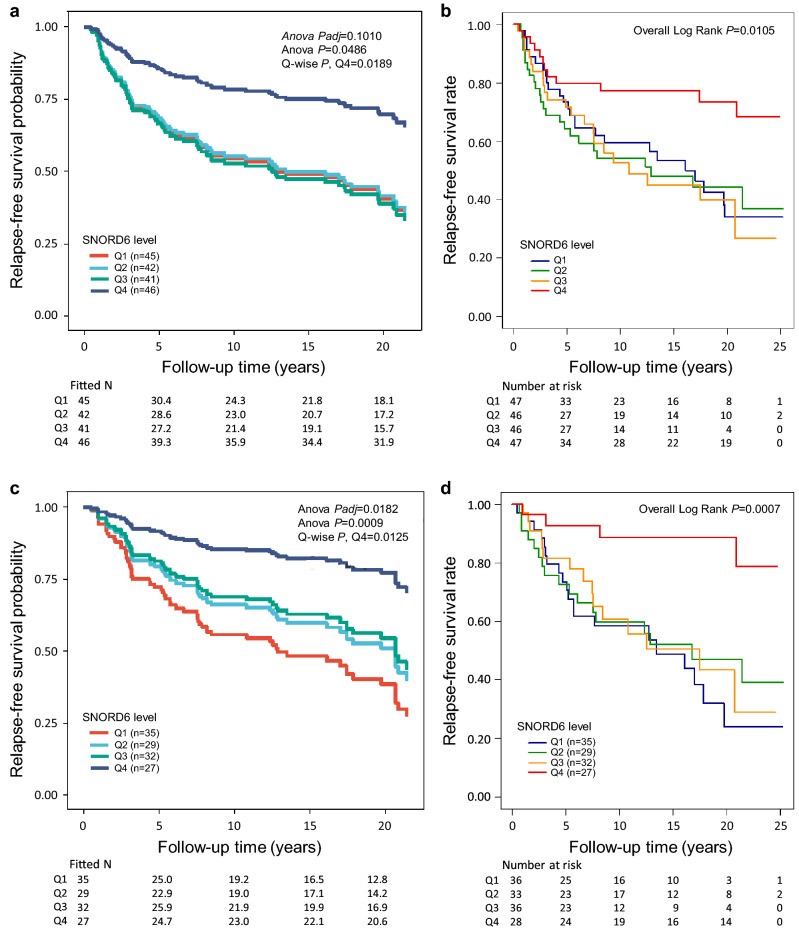
SNORD6 was identified as a prognostic marker in invasive local BC. (**a**) The highest quartile (Q4) of SNORD6 significantly associated with better RFS (Overall *Padj* = 0.1010, Overall *P* = 0.0486, for Q4 *P* = 0.0189, HR [CI 95%] = 0.43 [0.22–0.87]), when compared to the lowest quartile (Q1) in all cases with invasive local BC (n = 174) in the Cox multivariate survival analysis including the covariates tumor grade, tumor histology, tumor size, nodal status, ER status, PR status, HER2 status, age at diagnosis, and the treatment parameters radiotherapy (RT) (yes/no), adjuvant chemotherapy (CT) (yes/no), and adjuvant endocrine therapy (ET) (yes/no). Of the covariates, nodal status significantly associated with RFS in the multivariate analysis (Overall *P* = 5.405e − 05, for node positivity *P* = 0.0009, HR [CI 95%] = 2.17 [1.37–3.43). (**b**) Kaplan–Meier plot showing the association of SNORD6 with RFS in the univariate analysis (Overall Log Rank *P* = 0.0105) in all cases with invasive local BC (n = 186). (**c**) The highest quartile (Q4) of SNORD6 significantly associated with better RFS (Overall *Padj* = 0.0182, Overall *P* = 0.0009, for Q4 *P* = 0.0125, HR [CI 95%] = 0.24 [0.08–0.74]) also in ER positive cases (invasive local, n = 123) in the Cox multivariate analysis including covariates tumor grade, histology and size, nodal status, PR status, HER2 status, age at diagnosis, and the treatment parameters RT (yes/no), CT (yes/no), and adjuvant ET (yes/no). Of the covariates nodal status (Overall *P* = 0.0108, for node positivity *P* = 0.0027, HR [CI 95%] = 2.47 [1.37–4.46]), and age at diagnosis (Overall *P* = 0.0254, for age class ≤ 39 *P* = 0.0015, HR [CI 95%] = 4.51 [1.78–11.42]) significantly associated with RFS in the multivariate analysis. (**d**) Kaplan–Meier plot showing the association of SNORD6 with RFS in the univariate analysis (Overall Log Rank *P* = 0.0007) in the cases with invasive local, ER positive BC (n = 133). In (**a**) and (**c**), the fitted Ns were extrapolated from the multivariate-fitted survival probabilities.

Among the 42 sncRNAs, 23 associated with BC prognosis independently of the ER status of the tumors; the higher level of eight, four, and seven sncRNAs associated with poorer RFS, BCSS, and OS, respectively, whereas better RFS, BCSS, and OS were associated with the higher level of seven, six, and three sncRNAs, respectively. In many of the multivariate survival analyses also other (established) prognostic factors, including, typically, the nodal status, age at diagnosis, and tumor size, were significant. However, in some of the analyses the sncRNA was more significant than e.g. the forementioned. For example, longer RFS was observed with the patients with increased SNORD6 (ENSG00000202314.1) expression in all cases with ilBC in the multivariate analysis, while nodal status was a more significant factor than SNORD6 in the analysis (Fig. 4a, univariate analysis in Fig. 4b). In all ER positive ilBC cases SNORD6 was more significant than nodal status and age at diagnosis (RFS) in the multivariate analysis (Fig. 4c, univariate analysis in Fig. 4d), and in ER positive ilBC cases with only surgery SNORD6 alone significantly associated with RFS (Supplementary Fig. S15B).

**Figure 5:**
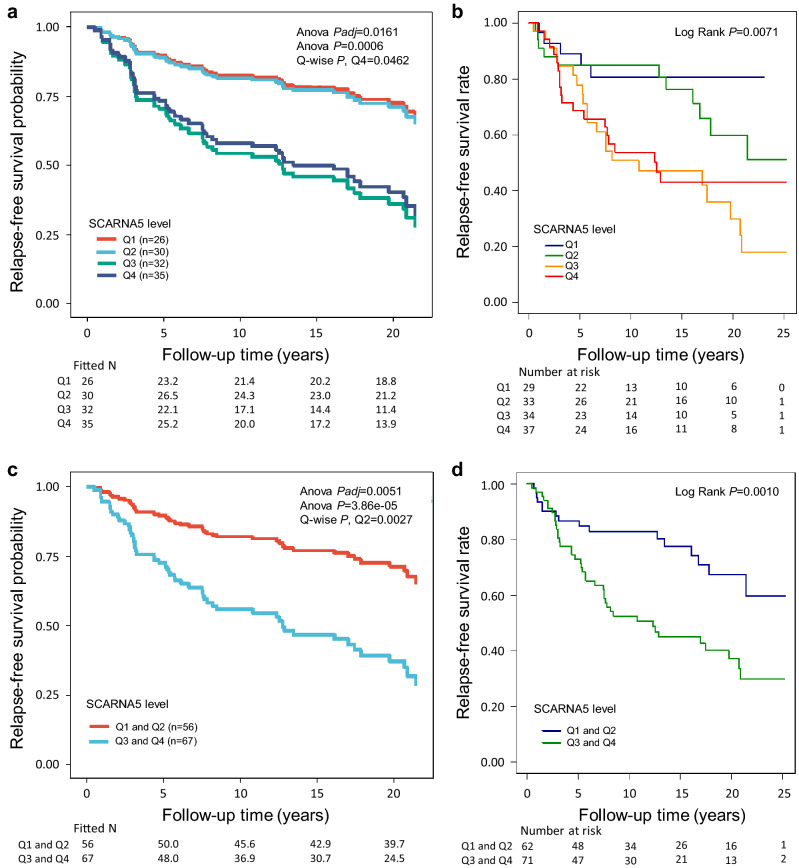
SCARNA5 showed prognostic potential in ER positive invasive local BC. (**a**) The highest quartile (Q4) of SCARNA5 significantly associated with poorer RFS (Overall *Padj* = 0.0161, Overall *P* = 0.0006, for Q4 *P* = 0.0462, HR [CI 95%] = 3.04 [1.02–9.08]), when compared to the lowest quartile (Q1) in ER positive cases (invasive local, n = 123) in the Cox multivariate analysis including the covariates tumor grade, histology and size, nodal status, PR status, HER2 status, age at diagnosis, and the treatment parameters RT (yes/no), CT (yes/no), and adjuvant ET (yes/no). Of the covariates nodal status (Overall *P* = 0.0096, for node positivity *P* = 0.0063, HR [CI 95%] = 2.35 [1.27–4.33]), and age at diagnosis (Overall *P* = 0.015, for age class ≤ 39 *P* = 0.0013, HR [CI 95%] = 4.68 [1.85–12.02]) significantly associated with RFS in the multivariate analysis. (**b**) Kaplan–Meier plot showing the association of SCARNA5 with RFS in the univariate analysis (Overall Log Rank *P* = 0.0071) in the invasive local, ER positive cases (n = 133). (**c**) The higher level of SCARNA5 associated with poorer RFS (Overall *Padj* = 0.0051, Overall *P* = 3.86e − 05, for higher half *P* = 0.0027, HR [CI 95%] = 2.79 [1.43–5.44]) in the cases with invasive local, ER positive BC also when the expression level was divided into two groups according to median in the Cox multivariate analysis including the covariates tumor grade, histology and size, nodal status, PR status, HER2 status, age at diagnosis, and the treatment parameters RT (yes/no), CT (yes/no), and adjuvant ET (yes/no). Also nodal status (Overall *P* = 0.0086, for node positivity *P* = 0.0061, HR [CI 95%] = 2.33 [1.27–4.28]), and age at diagnosis (Overall *P* = 0.014, for age class ≤ 39 *P* = 0.0010, HR [CI 95%] = 4.64 [1.86–11.61]) remained significant. (**d**) Kaplan–Meier plot showing the association of SCARNA5 with RFS according to median in the univariate analysis (Overall Log Rank *P* = 0.0010) in the cases with ER positive, invasive local BC (n = 133). In (**a**) and (**c**), the fitted Ns were extrapolated from the multivariate-fitted survival probabilities.

Of the 42 sncRNAs, 18 sncRNAs were found as candidate prognostic markers for ER positive ilBC as they associated with patient outcome only in the forementioned groups restricted to cases with ER positive ilBC (all ER positive cases, ER positive cases with only surgery, and/or RT-treated ER positive cases) (Supplementary Tables S10, S13, S15, Supplementary Fig. S15C). The higher level of seven sncRNAs associated with poorer RFS, and five with poorer BCSS. For example, SCARNA5 associated with poorer RFS only in all ER positive cases (i.e. when the analyses were not restricted to a specific treatment group), being a more significant factor in the Cox analysis (Overall *P*) than nodal status or age at diagnosis (Fig. 5a, univariate analysis in Fig. 5b). Similar association remained also when the SCARNA5 expression level was divided into two groups by median (Fig. 5c,d). The higher level of another seven, two, and one sncRNAs in turn associated with better RFS, BCSS, and OS.

Only one sncRNA (Y_RNA, ENSG00000199801.1) associated with patient outcome (poorer BCSS) only in the ER negative cases (Supplementary Table S11), the highest expression quartal having a stronger predictive effect in the Cox model (Overall *P* = 0.0130, for Q4 *P* = 0.0004, HR [CI 95%] = 8.12 [2.56–25.78]) than nodal status (Overall *P* = 0.0050, for node positivity *P* = 0.0053, HR [CI 95%] = 4.35 [1.55–12.26]). Tumor histology also remained statistically significant in the Cox analysis (Overall *P* = 0.0190, for lobular histology *P* = 0.0010, HR [CI 95%] = 51.04 [4.89–532.38], for ductal histology *P* = 0.0059, HR [CI 95%] = 12.70 [2.08–77.53]).

### Several sncRNAs predict response to RT

**Figure 6:**
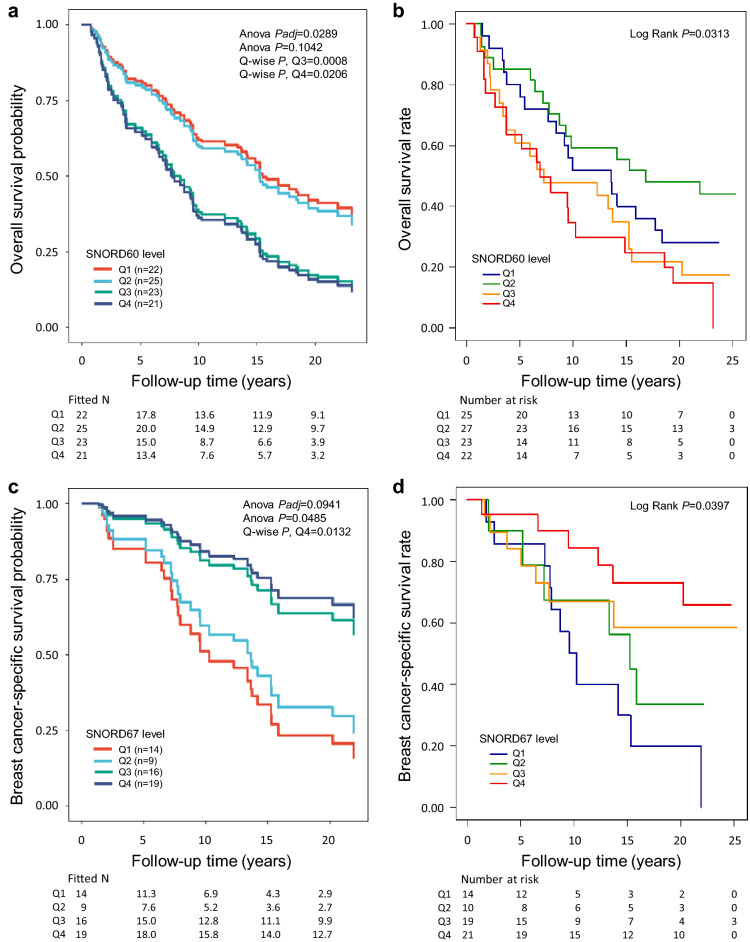
SNORD60 and SNORD67 were identified as candidates for predictive markers for RT in invasive local BC. (**a**) The higher quartiles (Q3 and Q4) of SNORD60 significantly associated with poorer OS in the Cox multivariate analysis including the covariates tumor grade, histology and size, nodal status, ER status, PR status, HER2 status, age at diagnosis, and the treatment parameters CT (yes/no), and adjuvant ET (yes/no) (Overall *Padj* = 0.1042, Overall *P* = 0.0289, for Q3 *P* = 0.0008, HR [CI 95%] = 3.90 [1.76–8.63], and for Q4 *P* = 0.0206, HR [CI 95%] = 2.48 [1.15–5.37]) in all cases with invasive local BC who had received RT (n = 91). (**b**) Kaplan–Meier plot showing the association of SNORD60 with OS in the univariate analysis (Overall Log Rank *P* = 0.0313) in all cases with invasive local BC who had received RT (n = 97). (**c**) The highest quartile (Q4) of SNORD67 significantly associated with better BCSS in the cases with ER positive BC (invasive local, n = 58) who had received RT (Overall *Padj* = 0.0941, Overall *P* = 0.0485, for Q4 *P* = 0.0132, HR [CI 95%] = 0.25 [0.08–0.75]; Cox multivariate analysis including the covariates tumor grade, histology and size, nodal status, PR status, HER2 status, age at diagnosis, and the treatment parameters CT (yes/no), and adjuvant ET (yes/no). (**d**) Kaplan–Meier plot showing the association of SNORD67 with BCSS in the univariate analysis (Overall Log Rank *P* = 0.0397) in cases with invasive local, ER positive BC who had received RT (n = 64). In (**a**) and (**c**), the fitted Ns were extrapolated from the multivariate-fitted survival probabilities.

Altogether 20 sncRNAs were identified to be predictive for RT response as they associated with patient outcome (Overall *P* < 0.05 and Q4 *P* < 0.05) only in the analyses including cases who had received RT (all, ER positive, or ER negative) or in addition in the analyses that were not restricted to any specific treatment group (all, ER positive, ER negative) (Supplementary Tables S14–S16, S9–S11, Supplementary Fig. S16). The higher level of six sncRNAs associated with poorer RFS, six with poorer BCSS, and seven with poorer OS. The higher level of four sncRNAs in turn associated with better RFS, six with better BCSS and five with better OS. For example, poorer OS was observed with the RT-treated cases with increased SNORD60 expression (Fig. 6a for multivariate and Fig. 6b for univariate analysis), whereas the increased SNORD67 expression associated with better BCSS in the ER positive RT-treated cases (Fig. 6c for multivariate and Fig. 6d for univariate analysis).

Additionally, the higher level of SNORD109B associated with better BCSS in the ER positive cases who had received RT and with better OS in the tamoxifen-treated ER positive cases indicating that SNORD109B might influence both RT and tamoxifen response in ER positive ilBC cases (Supplementary Tables S15, S17).

### A few sncRNAs show potential as predictive markers for tamoxifen response

**Figure 7:**
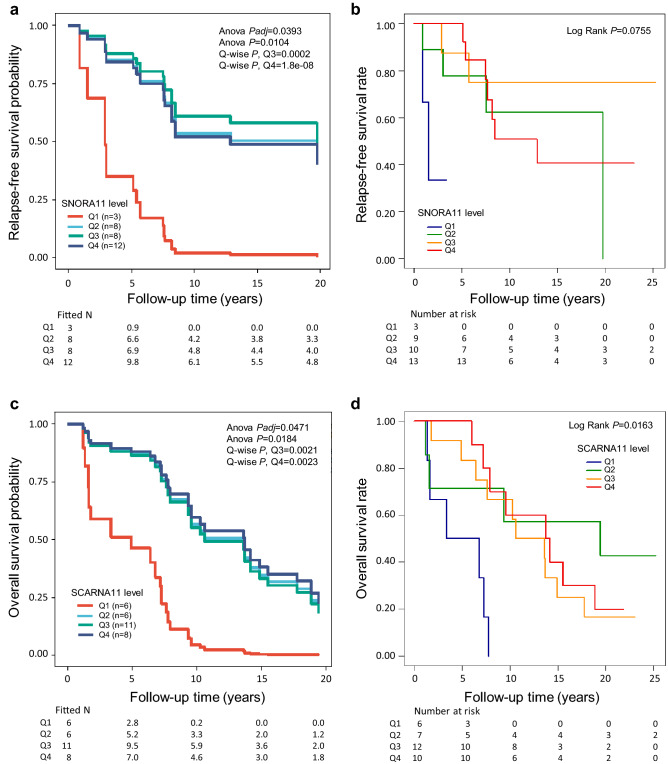
Examples of candidate sncRNAs predictive for tamoxifen response in invasive local BC. (**a**) The higher SNORA11 quartiles (Q3 and Q4) significantly associated with better RFS in the Cox multivariate analysis including the covariates tumor grade, histology and size, nodal status, PR status, HER2 status, age at diagnosis, and RT (yes/no) (Overall *Padj* = 0.0393, Overall *P* = 0.0104, for Q3 *P* = 0.0002, HR [CI 95%] = 0.05 [0.01–0.24], and for Q4 *P* = 1.8e − 08, HR [CI 95%] = 0.02 [0.01–0.08]), when compared to the lowest quartile (Q1) in the cases with ER positive BC who had received tamoxifen (invasive local, n = 31). (**b**) Kaplan–Meier plot showing the association of SNORA11 with RFS in the univariate analysis (Overall Log Rank *P* = 0.0755) in the tamoxifen-treated cases with invasive local, ER positive BC (n = 35). Note the low number of events in the Q1 group. (**c**) The higher SCARNA11 quartiles (Q3 and Q4) significantly associated with better OS in the Cox multivariate analysis including the covariates tumor grade, histology and size, nodal status, PR status, HER2 status, age at diagnosis, and RT (yes/no) (Overall *Padj* = 0.0471, Overall *P* = 0.0184, for Q3 *P* = 0.0021, HR [CI 95%] = 0.13 [0.04–0.48], and for Q4 *P* = 0.0023, HR [CI 95%] = 0.12 [0.03–0.47]), when compared to the lowest quartile (Q1) in the cases with ER positive BC who had received tamoxifen (invasive local, n = 31). (**d**) Kaplan–Meier plot showing the association of SCARNA11 with OS in the univariate analysis (Overall Log Rank *P* = 0.0163) in the tamoxifen-treated cases with local invasive, ER positive BC (n = 35). In (**a**) and (**c**), the fitted Ns were extrapolated from the multivariate-fitted survival probabilities. Note that the last time point in (**a**) is at 19.74 and in (**c**) 19.43 years.

Altogether eight sncRNAs associated with patient outcome (Overall *P* < 0.05 and Q4 *P* < 0.05) only in the ER positive cases who had received tamoxifen therapy suggesting they may influence tamoxifen response (Supplementary Table S17). The higher level of one sncRNA associated with poorer RFS, one with poorer OS, two with better RFS, and four with better OS. For example, the higher level of SNORA11 associated with better RFS (Fig. 7a for multivariate and Fig. 7b for univariate analysis), whereas the higher level of SCARNA11 associated with better OS (Fig. 7c for multivariate and Fig. 7d for univariate analysis). It should be taken into consideration that some of the tamoxifen response-associated sncRNAs were expressed at relatively low levels (Supplementary Table S2).

## Discussion

The growing indications of ncRNAs’ involvement in tumorigenesis have motivated cancer researchers to investigate them further. The sncRNAs have been suggested to have diverse roles in tumorigenesis and potential as diagnostic, prognostic as well as predictive markers. However, a considerable part of these studies has been conducted with small sample numbers and varying research protocols leading also to inconsistent results. Also, the high-throughput studies investigating the roles of non-miRNA/piRNA sncRNAs in BC account for the minority of the research conducted in this field. Therefore, we investigated the non-miRNA/piRNA sncRNA expression using small RNA-seq in our large material of fresh-frozen invasive BC and benign breast tissue samples.

Of the sncRNAs studied here, snoRNAs are the most extensively evaluated sncRNAs in cancer and thus functional mechanisms have already been suggested for their possible involvement in tumorigenesis^17^. We identified 58 snoRNAs/scaRNAs distinguishing cancerous tissue from benign breast tissue, suggesting their involvement in the development of invasive BC and possibilities as cancer biomarkers. The differential expression of multiple snoRNAs e.g. the upregulation of SNORD41, SNORD83A, and SNORA73B, and the downregulation of SNORD59A, SNORD111B, and SNORD119 detected in our samples has been previously observed in BC^25,26^. Additionally, the prominent downregulation of SNORD113/SNORD114 cluster seen here has been reported in TNBC and in colorectal cancer when compared to adjacent normal tissue^41,42^. We also identified four snoRNAs that significantly distinguished metastasized BC from ilBC. To our knowledge, the association between these four snoRNAs and metastasis has not been previously reported, although, SNORD104, SNORD105, and SNORD82 have been suggested as prognostic markers for BC^25^. Further research on the mechanisms through which the identified snoRNAs, especially SNORD104, could affect cancer spreading is required.

We found 51 snoRNAs/scaRNAs associated with the clinicopathological characteristics of BC including tumor grade, the hormone receptor status of the tumors, molecular subtype, and tumor histology offering further candidates for prognostic and therapeutic markers for ilBC. Various snoRNAs/scaRNAs associated with the tumor grade, a universally recognized prognostic determinant, including several snoRNAs/scaRNAs that associated with the tumor grade independently of the tumors’ ER status. A few of the benign breast tissue-associated snoRNAs from SNORD113/114 cluster (see above) were also upregulated in lower grade malign tumors highlighting their possible significance in BC development. Eleven snoRNAs/scaRNAs were identified as characteristic to TNBC and 25 to luminal BC. Only SNORD99 associated with tumor histology being upregulated in ductal compared to lobular carcinoma, two main histological types of BC that differ in clinicopathological characteristics and therapy responsiveness^43^. The upregulation of SNORD99 was observed also in higher grade tumors, ER negative tumors, and in TNBC. Interestingly, the overexpression of its host gene *SNHG12* has been indicated to be mediated by c-MYC resulting in aggressive phenotype in MDA-MB-231 and BT-549 cells (TNBC cell lines)^44^. Supporting our findings, the expression of the here observed TNBC-associated SNORD99 and SNORD93 has previously been detected in TNBC, and SNORD93-derived RNA expression also in MDA-MB-231 cells in which its upregulation associated with enhanced invasiveness, a typical characteristic of the cell line^45,46^. Moreover, we found a previously reported HER2-type BC-associated SNORD124 to be the only sncRNA that associated with the HER2 receptor status^47^.

The TNBC-associated SNORD92 and SNORD72 were upregulated also in the ER negative and PR negative tumors, whereas the luminal BC-associated SNORA11 and SNORD104 associated also with ER positive and PR positive tumors in our samples. All these four snoRNAs associated with the tumor grade and three of them also with patient outcome indicating their possible role in BC tumorigenesis. SNORA11 seemed to display a protective effect in ilBC for it associated with better outcome, lower grade, luminal BC, and the receptor positive status in our samples. Given their observed prominent associations with multiple clinicopathological features of BC, SNORD99, SNORD92, SNORD72, SNORA11, and SNORD104 were among the most intriguing candidates identified here. Previously the upregulation of SNORD72 has been reported to distinguish cancer from normal counterparts and to promote liver cancer cell invasiveness via stabilizing *ID2* mRNA, whereas SNORD92 has been linked to BC patient outcome^25,26,48,49^.

Multiple snoRNAs/scaRNAs were identified as candidates for prognostic markers for ilBC, several of which associated with patient outcome only in the ER positive cases (all and/or surgery only), which nominates these as candidates for prognostic markers for ER positive BC. Many of these snoRNAs/scaRNAs have been previously reported in cancer providing evidence of their possible involvement in tumorigenesis^24,26,29,42,49^. Gong et al. observed the clinical relevance of SNORD88A, SCARNA5, SNORD11, and SNORD12C in various cancer types including BC in the vast TCGA material, and other studies have suggested SNORA80E as a marker for poorer cancer prognosis, thus supporting our findings^26,50–52^. In line with the here observed association of SNORD105B with ER negative tumors, TNBC, and poorer outcome, its upregulation has been detected in colon adenocarcinoma (CoAC) and its oncogenic role in promoting tumor growth and invasiveness possibly by interacting with ALDOA indirectly affecting *c-MYC* expression was described in gastric cancer^49,53^. We also observed a clear association with poorer patient outcome with higher SNORA65 and SNORD3C, and better outcome associating with higher SNORD6. To our knowledge, their prognostic potential in BC has not been previously described. However, the upregulation of NOP10, a vital part of the H/ACA snoRNP complex, has been linked to poorer outcome of lung cancer patients and its depletion resulted in the decreased level of a few snoRNAs including SNORA65, leading to impaired tumor growth^54^. We saw SNORD3C associating also with invasive BC and SNORD6 with benign breast tissue which supports our observations from the survival analyses. The functional characterization of especially SNORD6 and SCARNA5 in BC could provide information on BC progression.

Choosing the most appropriate therapeutic modality at the time of diagnosis would improve the chance of survival of BC patients. Therefore, we looked for candidate predictive markers and observed several snoRNAs/scaRNAs that may influence the efficacy of RT or tamoxifen therapy. In our study, SNORD82 associated with better outcome in cases who had received RT and was also upregulated in benign breast tissue compared to ilBC, and in ilBC compared to metastasized BC, thus suggesting it could be indicative of a less aggressive phenotype. The clinical relevance and prognostic potential of SNORD82 in cancer including BC has been previously reported^25,26^. The here observed markers for better therapy response, SNORD66 and SCARNA11 have been reported to be upregulated in cancer tissue compared to the corresponding normal tissue, and SNORD67 has been linked to better outcome also in COAC^21,29,49^. Consistently, SNORD66 and SCARNA11 associated also with ER positive tumors and luminal BC, and SNORD66 additionally with lower grade tumors in our samples. Additionally, the higher expression of SNORD60, a novel candidate for cancer prognosis, clearly correlated with poorer OS in the RT-treated cases. Since the higher levels of SNORD66 have been correlated with poorer OS in non-small-cell lung carcinoma, and the high sdRNA level of the here identified poorer outcome-associated SNORA77 has been observed to have different survival rates depending on cancer type, they may have tissue-specific effects in cancer^21,55^. Based on our results, we nominated SNORD67, SNORD60, and SCARNA11 as the most prominent predictive candidates for invasive local BC.

Given the vast influence of alternative splicing on gene regulation, it is not surprising that splicing factors have recently been implicated to affect tumorigenesis^19,56^. Particularly, the fragments of U2 snRNA have been suggested to have diagnostic and prognostic value in different cancer types^23,57,58^. We observed the differential expression of ten snRNAs, including the higher level of U2, in ilBC compared to benign breast tissue. The ilBC-associated RNU2-36P and RNU4-1 showed upregulation also in the higher grade tumors and tumors with receptor negative status. RNU2-36P additionally associated with TNBC, and with higher grade independently of the ER status, thus making it the most intriguing DE snRNA in our samples. Supporting our results, the presence of RNU2-36P and RNU7-3P has been previously observed in TNBC, and the upregulation of RNU2-36P also in adrenocortical carcinoma^45,59^. We identified a few snRNAs also as candidates for prognostic markers for ilBC. The higher levels of RNU5A-1 and RNU4-2 associated with poorer prognosis and have been previously reported in colon cancer tissue compared to normal tissue^60^. We saw the upregulation of RNU4-2 in ilBC when compared to benign breast tissue in our samples. RNU5A-1 and RNU4-1 have been linked to patient outcome-associated alternative splicing in endometrial cancer^61^.

The described dysregulation of Y RNAs and Y RNA-derived fragments in multiple cancer types and their introduced roles in apoptosis, stress response, and cell proliferation suggests their participation in tumorigenesis^18^. In addition, VTRNAs have been indicated to participate in tumor-associated functions such as apoptosis and drug resistance^20,62,63^. We found 28 miscRNAs distinguishing ilBC from benign breast tissue. The here observed upregulation of RNY1 and RNY4 in BC is consistent with previous reports^27,64^. Additionally, a few miscRNAs were identified as characteristic to the molecular subtype of BC. The TNBC-associated RNY4 and VTRNA1-1 were upregulated also in ER and in PR negative tumors, whereas VTRNA1-2 associated also with ER negative tumors. All these three miscRNAs and an additional TNBC-associated miscRNA, RNY1, were also upregulated in ilBC when compared to benign breast tissue. VTRNA1-1 was additionally upregulated in higher grade tumors and ductal carcinoma. As the here identified candidates VTRNA1-1, RNY1, and RNY4 have been suggested to distinguish cancer from normal counterparts^24,27,28,42^, and VTRNA1-1 also to inhibit apoptosis and affect chemoresistance^62,63,65^, they could have roles in the mechanisms that are involved also in the aggressiveness of TNBC. We observed RN7SL508P and RNY1 as candidates for markers of better prognosis for ilBC, RNY1 specifically for ER positive BC. Taken together, especially VTRNA1-1, RNY4, and RNY1 showed potential as diagnostic/prognostic candidates for ilBC. Since the number of tamoxifen-treated cases was quite low in our analyses and the predictive potential of the here identified miscRNAs (RN7SL1 and Y_RNA ENSG00000201800) have not been previously reported, they require further evaluation.

Here we have found altogether 71 sncRNAs associated with patient outcome, of which 25 associated also with one or more of the clinicopathological characteristics of ilBC. The identification of novel therapeutic targets could revolutionize the clinical handling of BC and would help conquer the challenges related to the lack of effective therapies against the aggressive and metastasis-prone TNBC, the variation of outcomes within a BC subtype, and the intrinsic and gained resistance to therapies^66–68^. It remains to be investigated whether, and how the subtype-associated sncRNAs identified here participate in the processes related to the aggressiveness of TNBC. Also, the sncRNAs identified as candidate prognostic markers for ER positive BC or as predictive markers may aid in distinguishing the ER positive cases with poorer outcome and provide knowledge of the mechanisms behind the insensitivity to RT and tamoxifen, and thus after further evaluation of their biological significance may offer targets for novel therapies. The majority of the snoRNAs identified here target the 18S or 28S rRNAs and might therefore affect tumorigenesis by influencing the translation of cancer-associated genes through ribosome alterations^26,54,69^. Additionally, they may participate in alternative splicing and gene regulation by stabilizing mRNAs or suppressing genes in a miRNA-like manner^46,48,53^. The identified candidate snRNAs and miscRNAs may in turn contribute to tumorigenesis by affecting alternative splicing (snRNAs), promoting DNA replication, and cell cycle progression (Y RNAs), preventing cancer cells from undergoing apoptosis, and inflicting drug resistance (VTRNAs)^18–20,62,63^. As Y RNAs associate with various RNA-stabilizing proteins they could also have roles in regulating gene expression^18^. Although the precise mechanisms through which the here identified candidate sncRNAs could function in BC remain to be further investigated, many of the candidates have been previously linked to cancer, and even functional roles have been introduced for some, suggesting their involvement in tumorigenesis.

To our knowledge, this is the first high-throughput study to investigate the associations of snoRNAs, snRNAs, and miscRNAs with various clinicopathological characteristics of BC in a large number of fresh-frozen tissue samples, and patient survival with long follow-up time. We identified several sncRNAs as candidates for novel markers of invasive BC and paved the way for intriguing opportunities for future research to determine their possible functional roles in BC, which could provide novel strategies for BC treatment over time.

## Supplementary Information

Supplementary Information 1.

Supplementary Information 2.

Supplementary Information 3.

Supplementary Information 4.

Supplementary Information 5.

Supplementary Information 6.

Supplementary Information 7.

Supplementary Information 8.

Supplementary Information 9.

Supplementary Information 10.

Supplementary Information 11.

Supplementary Information 12.

Supplementary Information 13.

Supplementary Information 14.

Supplementary Information 15.

Supplementary Information 16.

Supplementary Information 17.

Supplementary Information 18.

## Data Availability

The study participant consent protects the privacy of the patients and does not allow opening the sequencing data generated and analyzed during the current study, but they are available from the corresponding author [J.M.H.] on reasonable request. The vst-normalized expression for those 1949 genes that had any reads in any sample, and key clinical parameters for all 228 samples are available as Supplementary Table S2.
