# Supplementary material for: Expression profiles of small non-coding RNAs in breast cancer tumors characterize clinicopathological features and show prognostic and predictive potential
Source: Sci Rep. 2022 Dec 30;12:22614. doi: 10.1038/s41598-022-26954-w (PMC9803687; doi:10.1038/s41598-022-26954-w)
Supplement: Supplementary file 1 — Supplementary Information 1. [file 41598_2022_26954_MOESM1_ESM.pdf]

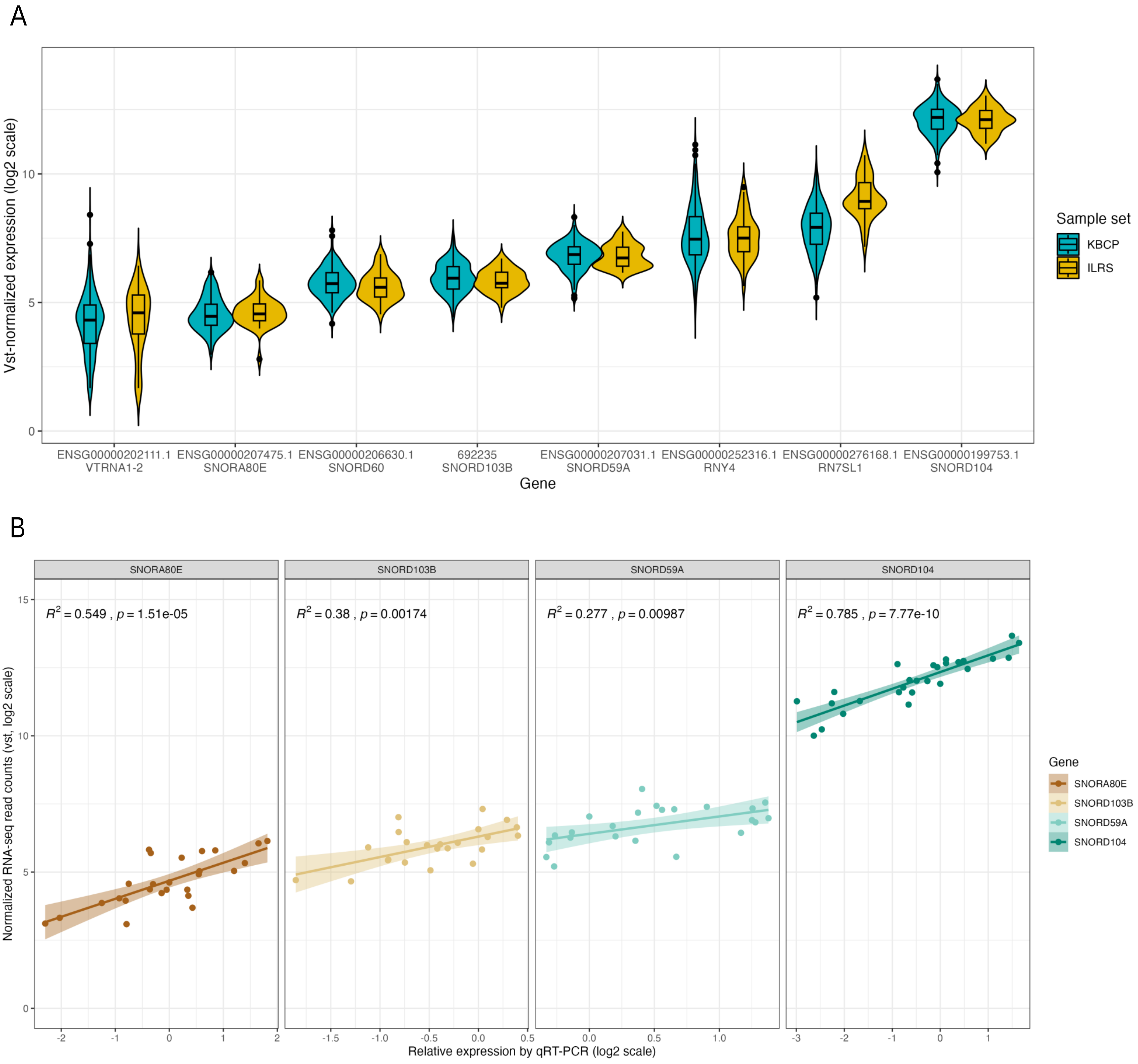

Supplementary Figure S1. Validation of RNA-seq results. **A)** Comparison of the expression of the top DE sncRNAs in invasive BC tumors from KBCP and ILRS* material. The violin plot of vst-normalized gene expression in invasive KBCP (N=186) and ILRS (N=40) breast cancers shows the similarity of the median expression as well as the shape of distribution between the two cohorts for all the included 8 DE sncRNAs. **B)** Relative expression measured using qRT-PCR on x-axis is plotted against vst-normalized RNA-seq read counts on y-axis, both in log2 scale, for four indicated RNAs. The plots include a linear regression line with the surrounding colored area representing 95% confidence interval. The Pearson correlation R^2 and two-sided *P*-values are given at the top left corners of each plot.

*The ILRS Project is an additional prospective, population-based collection of BC cases that has been collected in KUH during 2010-2014. Similar to KBCP, ILRS includes systematically collected tissue samples and data of biological, up-to-date therapeutic and environmental factors from BC cases diagnosed and treated at KUH. The ILRS data (or analyses) from small RNA-sequencing have not been published yet but were used here for validating the expression of the selected sncRNAs (SNORA80E [ENSG00000207475.1], SNORD103B [NCBI: 692235], SNORD59A [ENSG00000207031.1], SNORD104 [ENSG00000199753.1], SNORD60 [ENSG00000206630.1], RN7SL1 [ENSG00000276168.1], VTRNA1-2 [ENSG00000202111.1], and RNY4 [ENSG00000252316.1]) in a second cohort of invasive local BC cases (34 ductal and 6 lobular BCs; 4 ER negative and 36 ER positive).

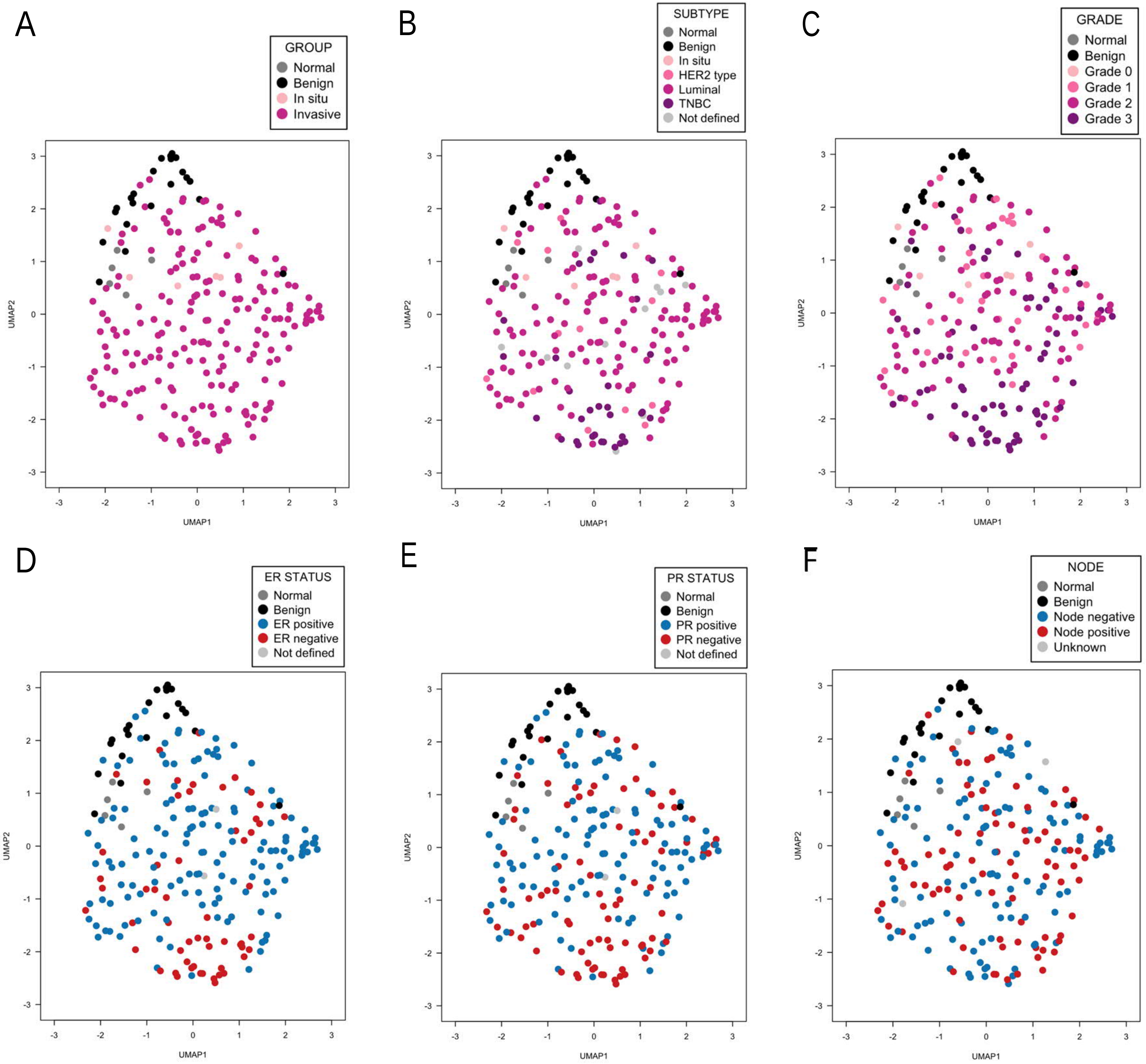

Supplementary Figure S2. Unsupervised clustering using uniform manifold approximation and projection (UMAP) suggests differences between some, but not all clinical parameters. All the 228 samples were clustered using UMAP based on the 219 top expressed and variable sncRNAs, plotting the first 2 UMAP components and coloring the samples for **A**) the main sample group, **B**) the sample subtype, **C**) tumor grade, **D**) ER status, **E**) PR status, and **F**) nodal status, as indicated. Not defined, insufficient information to define the non-normal, non-benign status or subtype, or receptor status; unknown, nodal status unknown.

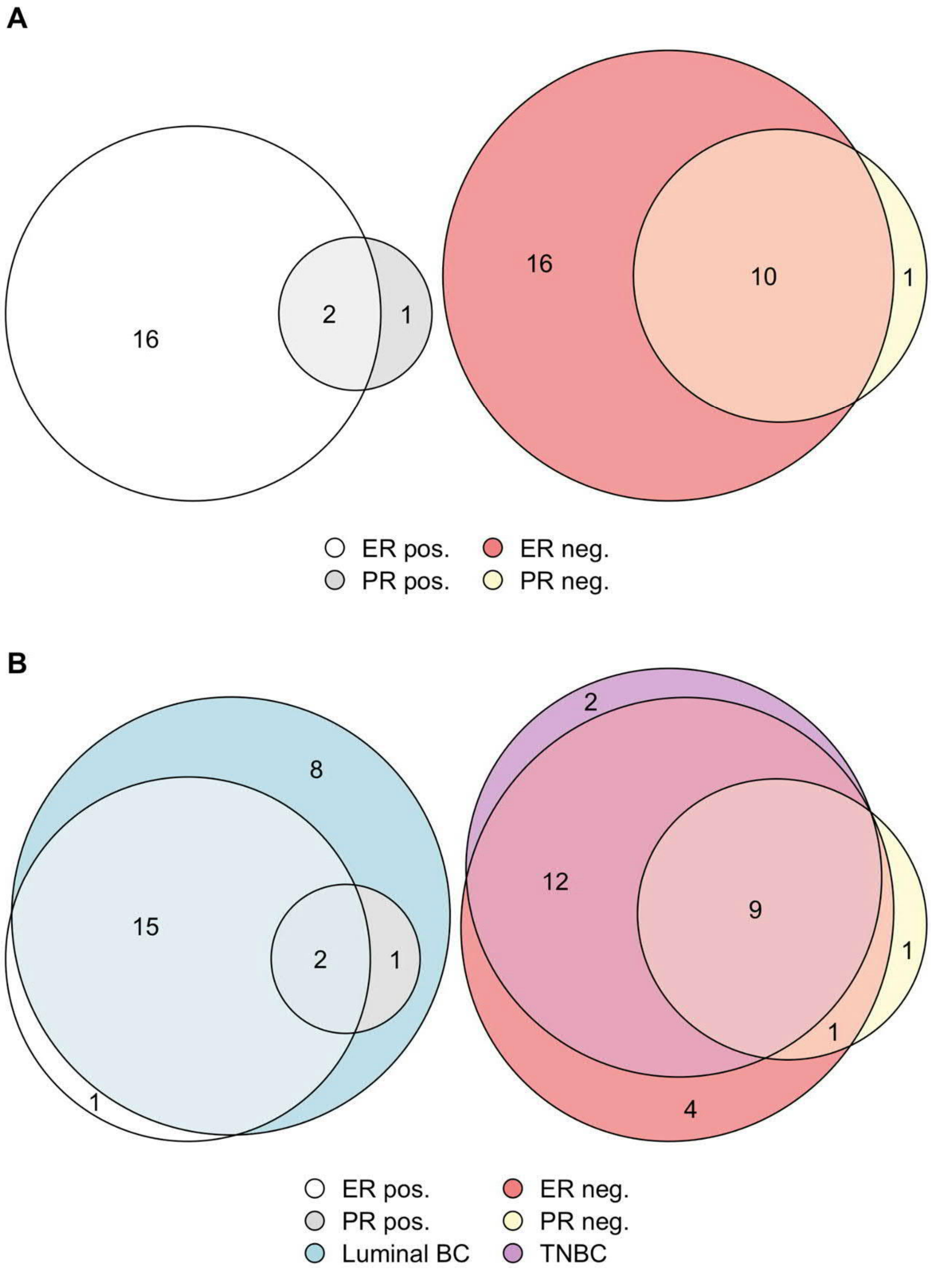

Supplementary Figure S3. The association of tumor hormone receptor status and molecular subtype with the sncRNA expression. Euler diagrams presenting the number and overlap of DE sncRNAs upregulated in **A)** ER or PR negative, and ER or PR positive BC, and **B)** ER or PR negative and TNBC, and ER or PR positive and luminal BC.

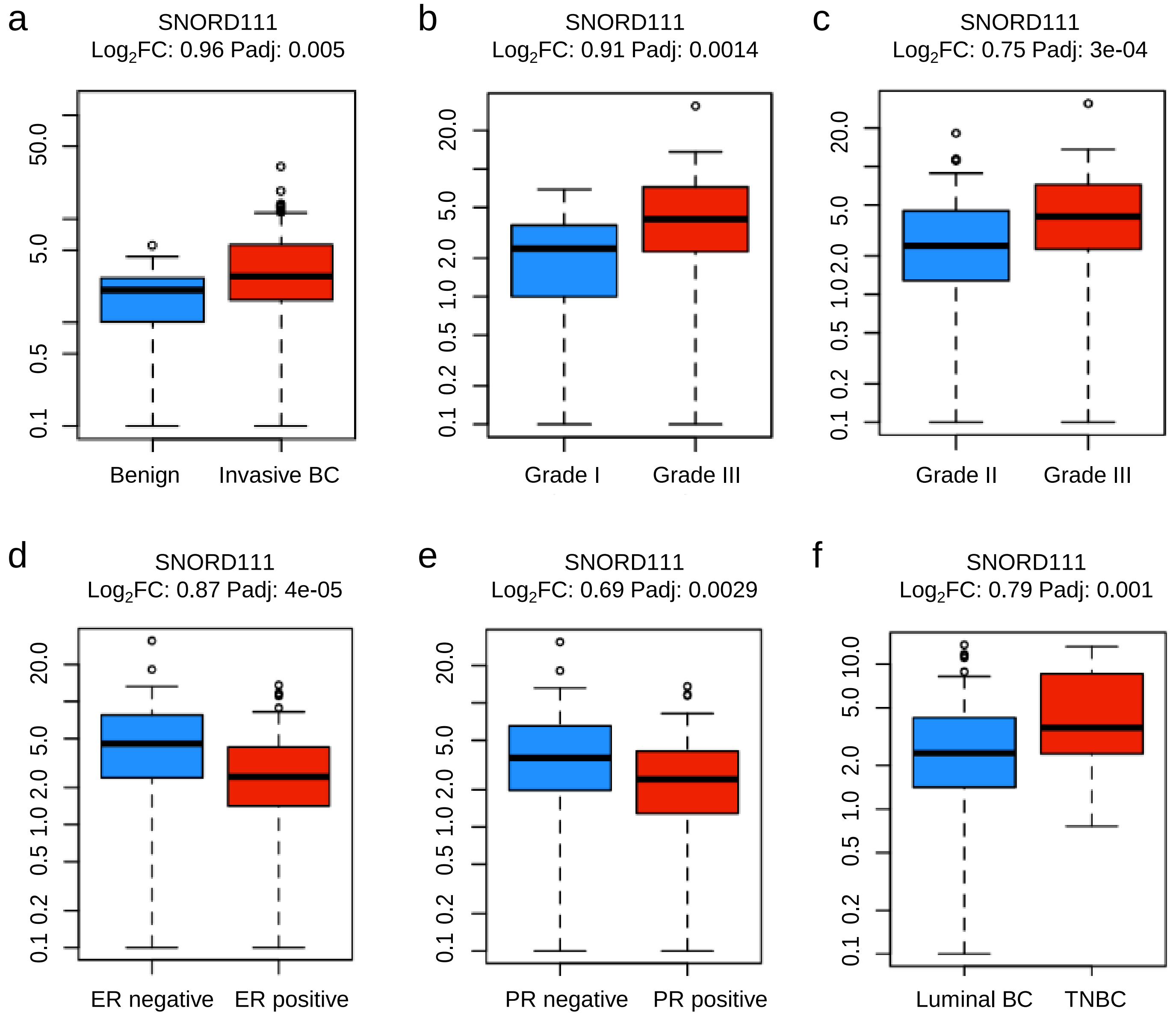

Supplementary Figure S4. The significant associations (*Padj*<0.01) of SNORD111 with the clinicopathological features of invasive breast cancer (BC). (**a**) SNORD111 was upregulated in invasive local BC vs. benign breast tissue, (**b**) in grade III tumors vs. grade I tumors, (**c**) in grade III vs. grade II tumors, (**d**) in estrogen receptor (ER) negative tumors vs. ER positive tumors, (**e**) in progesterone receptor (PR) negative tumors vs. PR positive tumors, and (**f**) in triple-negative BC (TNBC) vs. luminal BC.

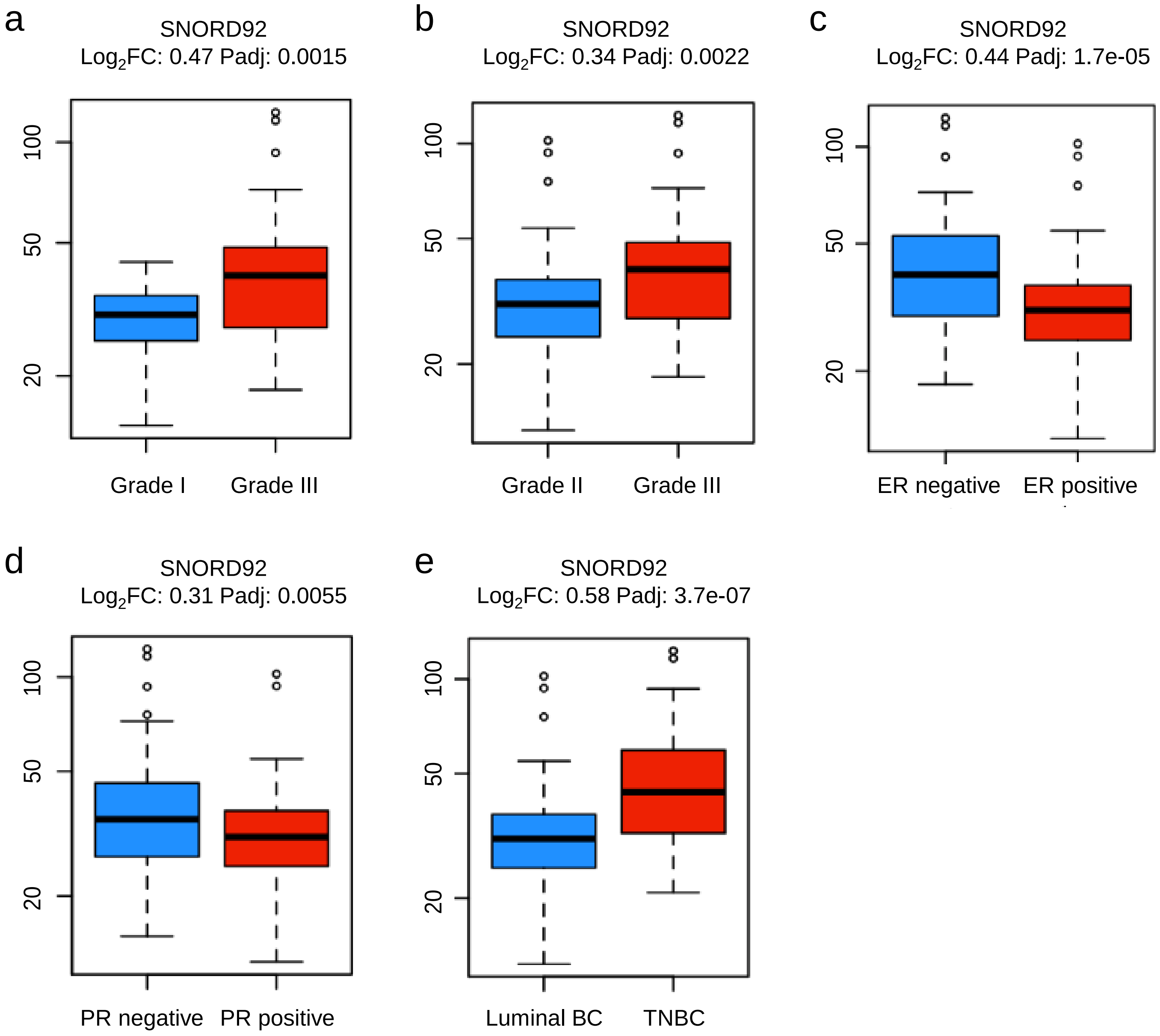

Supplementary Figure S5. The significant associations (*Padj*<0.01) of SNORD92 with the clinicopathological features of invasive breast cancer (BC). (**a**) SNORD92 was upregulated in grade III tumors vs. grade I tumors, (**b**) in grade III vs. grade II tumors, (**c**) in estrogen receptor (ER) negative tumors vs. ER positive tumors, (**d**) in progesterone receptor (PR) negative tumors vs. PR positive tumors, and (**e**) in triple-negative BC (TNBC) vs. luminal BC.

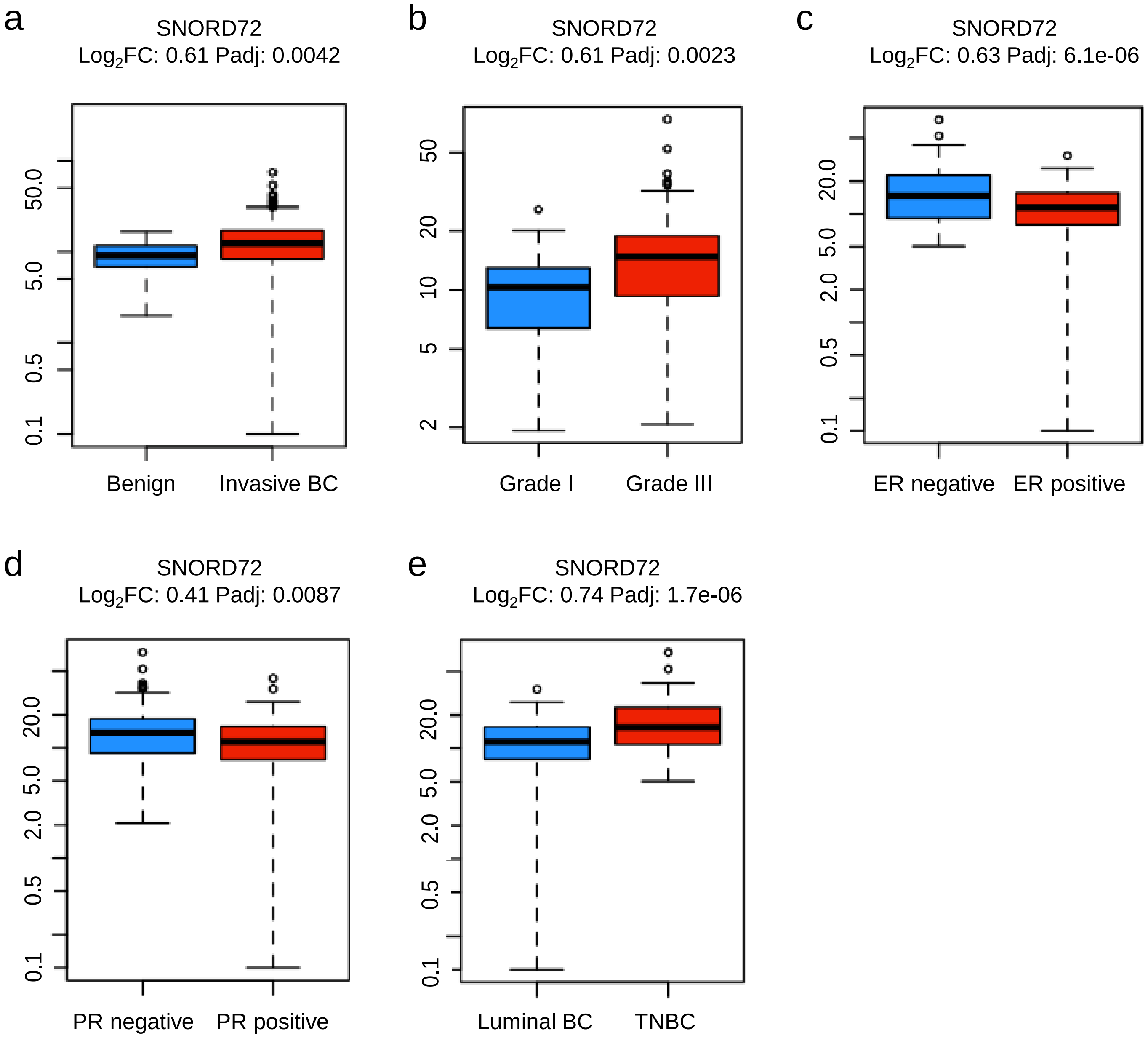

Supplementary Figure S6. The significant associations (*Padj*<0.01) of SNORD72 with the clinicopathological features of invasive breast cancer (BC). (**a**) SNORD72 was upregulated in invasive local BC vs. benign breast tissue, (**b**) in grade III vs. grade I tumors, (**c**) in estrogen receptor (ER) negative tumors vs. ER positive tumors, (**d**) in progesterone receptor (PR) negative tumors vs. PR positive tumors, and (**e**) in triple-negative BC (TNBC) vs. luminal BC.

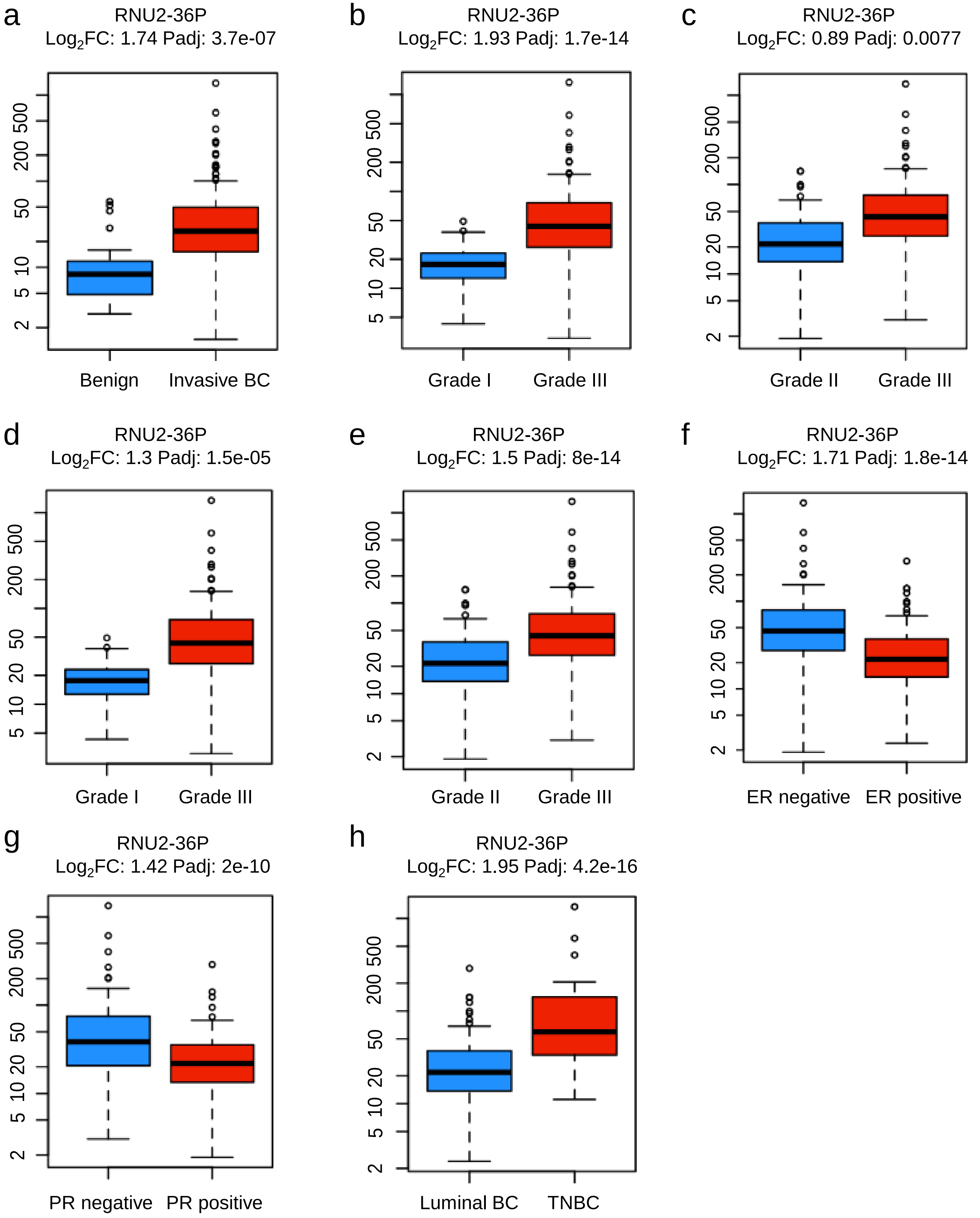

Supplementary Figure S7. The significant associations (*Padj*<0.01) of RNU2-36P with the clinicopathological features of invasive breast cancer (BC). (**a**) RNU2-36P was upregulated in invasive local BC vs. benign breast tissue, (**b**) in grade III vs. grade I tumors, (**c**) in grade III tumors vs. grade II tumors, (**d**) in grade III tumors vs. grade I tumors independently of the estrogen receptor (ER) status of the tumors, (**e**) in grade III tumors vs. grade II tumors independently of the ER status of the tumors, (**f**) in ER negative tumors vs. ER positive tumors, (**g**) in progesterone receptor (PR) negative tumors vs. PR positive tumors, and (**h**) in triple-negative BC (TNBC) vs. luminal BC.

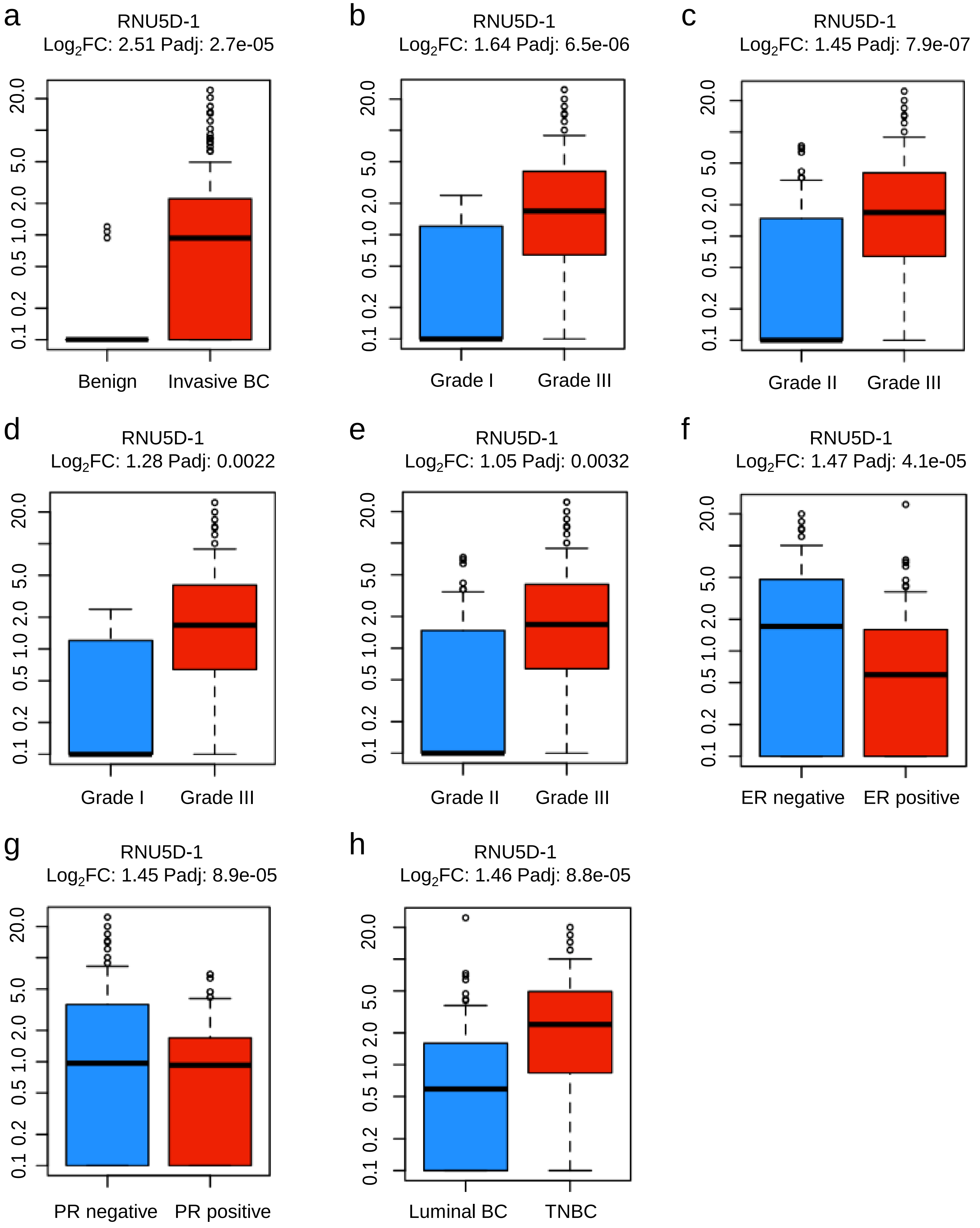

Supplementary Figure S8. The significant associations (*Padj*<0.01) of RNU5D-1 with the clinicopathological features of invasive breast cancer (BC). RNU5D-1 was upregulated (**a**) in invasive local BC vs. benign breast tissue, (**b**) grade III tumors vs. grade I tumors, (**c**) in grade III vs. grade II tumors, (**d**) in grade III tumors vs. grade I tumors independently of the estrogen receptor (ER) status of the tumors, (**e**) in grade III tumors vs. grade II tumors independently of the ER status of the tumors (**f**) in ER negative tumors vs. ER positive tumors, (**g**) in progesterone receptor (PR) negative tumors vs. PR positive tumors, and (**h**) in triple-negative BC (TNBC) vs. luminal BC.

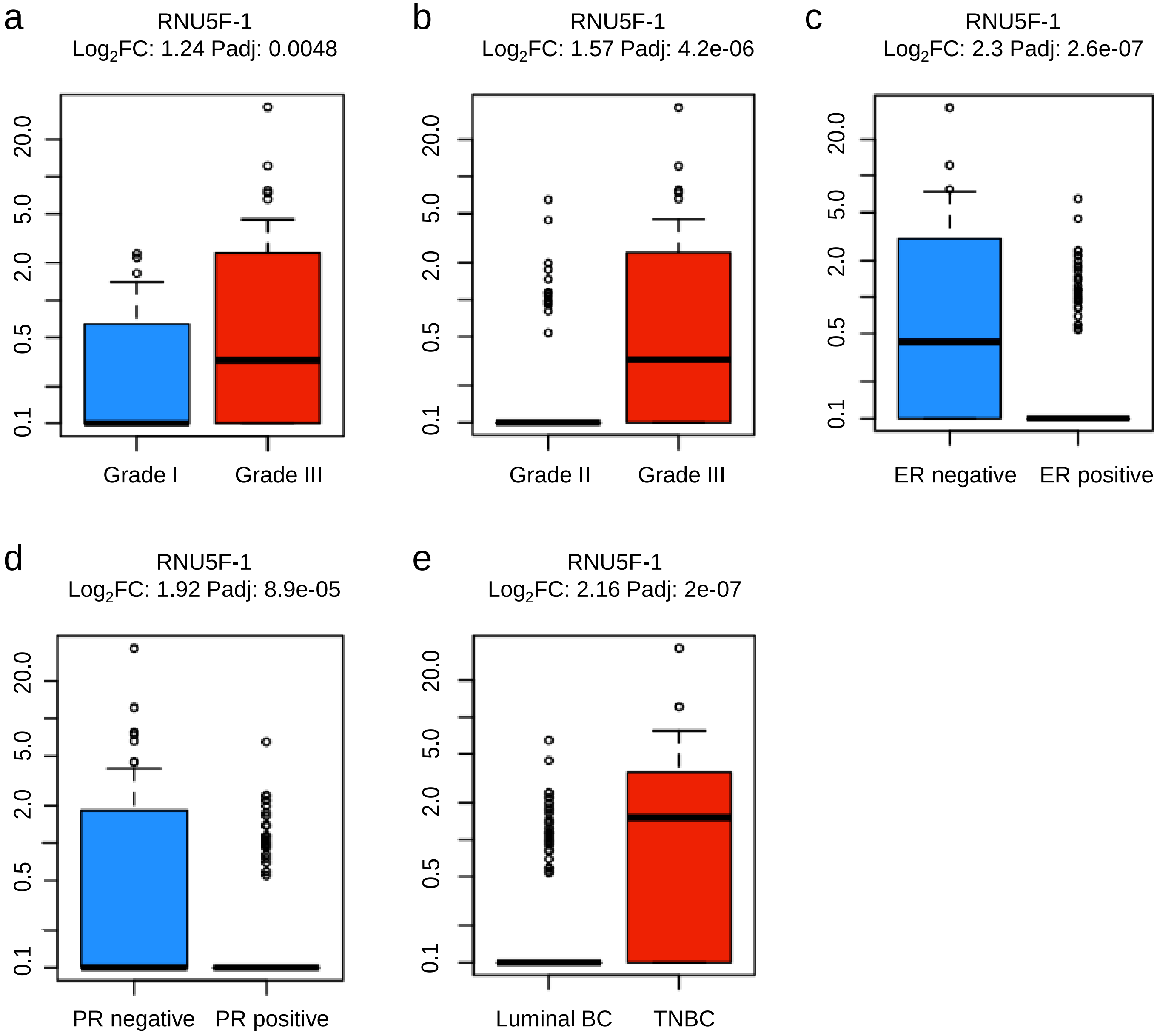

Supplementary Figure S9. The significant associations (*Padj*<0.01) of RNU5F with the clinicopathological features of invasive breast cancer (BC). RNU5F-1 was upregulated (**a**) grade III tumors vs. grade I tumors, (**b**) in grade III vs. grade II tumors, (**c**) in estrogen receptor (ER) negative tumors vs. ER positive tumors, (**d**) in progesterone receptor (PR) negative tumors vs. PR positive tumors, and (**e**) in triple-negative BC (TNBC) vs. luminal BC.

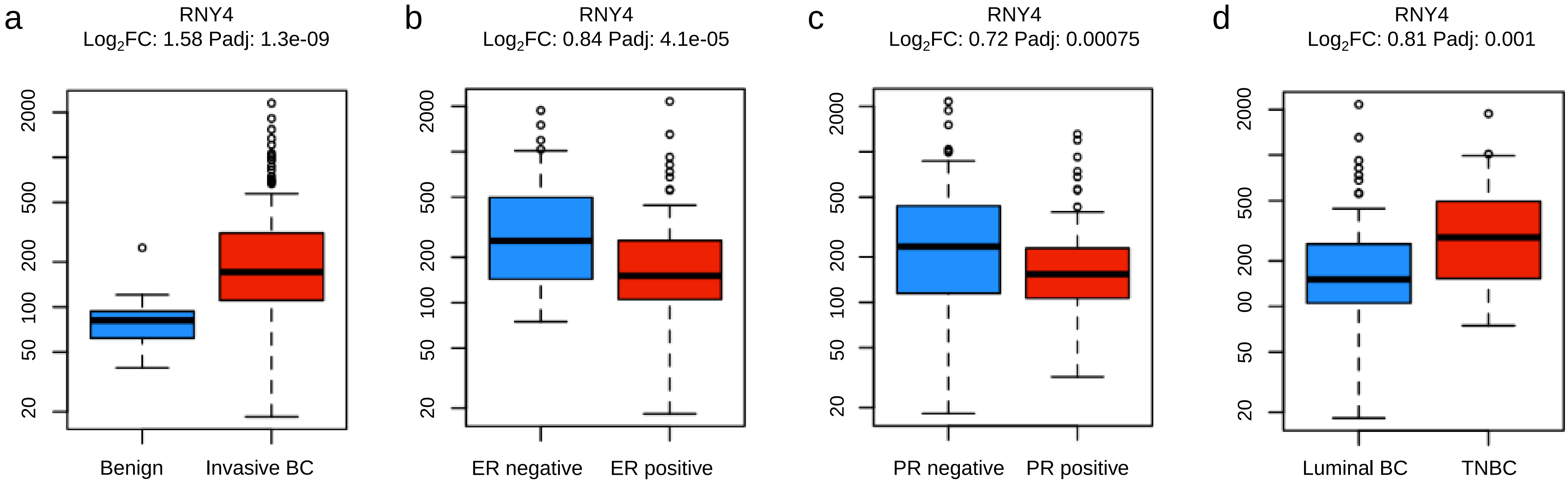

Supplementary Figure S10. The significant associations (*Padj*<0.01) of RNY4 with the clinicopathological features of invasive breast cancer (BC). RNY4 was upregulated (**a**) in invasive local BC vs. benign breast tissue, (**b**) in estrogen receptor (ER) negative tumors vs. ER positive tumors, (**c**) in progesterone receptor (PR) negative tumors vs. PR positive tumors, and (**d**) in triple-negative BC (TNBC) vs. luminal BC.

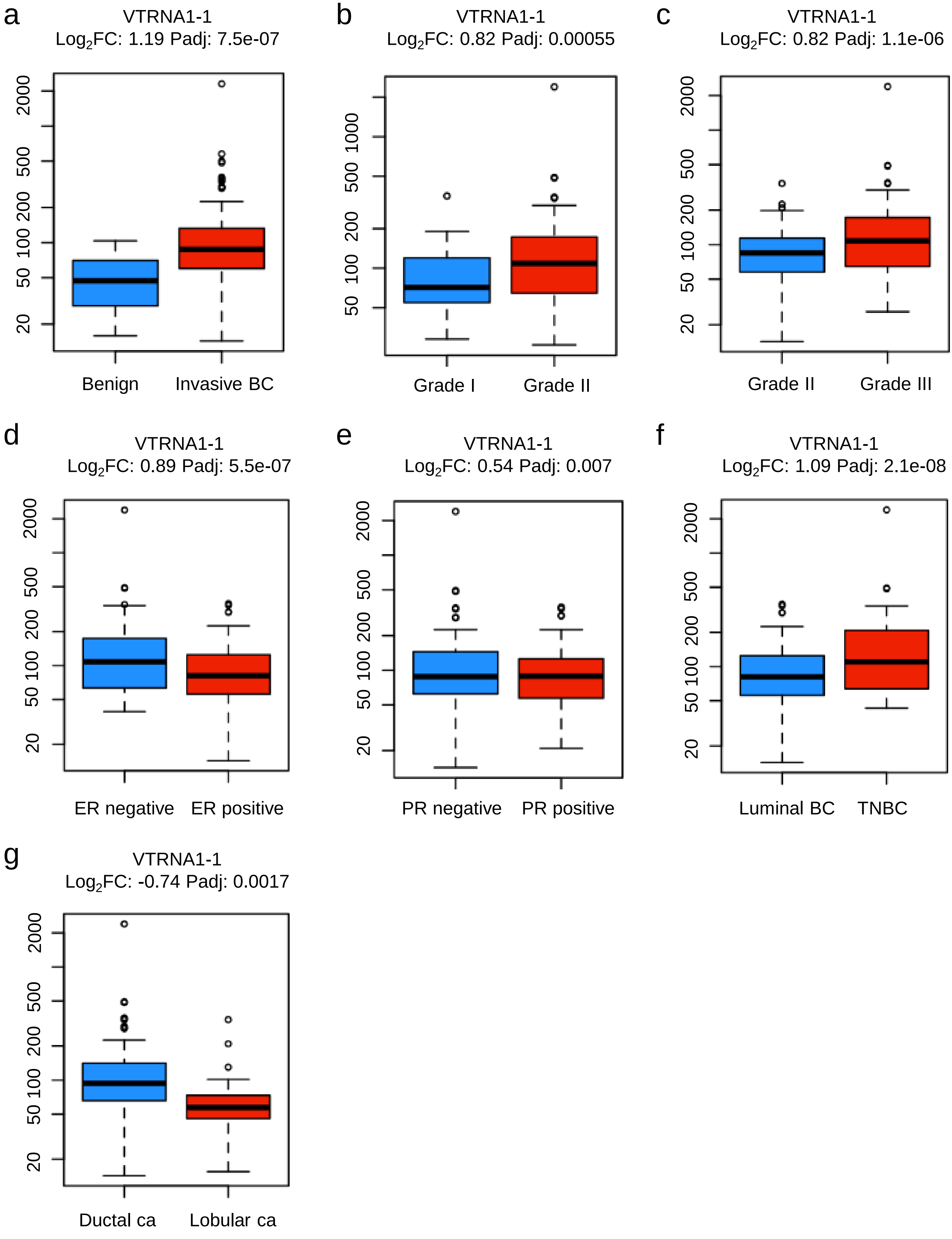

Supplementary Figure S11. The significant associations (*Padj*<0.01) of VTRNA1-1 with the clinicopathological features of invasive breast cancer (BC). VTRNA1-1 was upregulated (**a**) in invasive local BC vs. benign breast tissue, (**b**) grade III tumors vs. grade I tumors, (**c**) in grade III vs. grade II tumors, (**d**) in estrogen receptor (ER) negative tumors vs. ER positive tumors, (**e**) in progesterone receptor (PR) negative tumors vs. PR positive tumors, (**f**) in triple-negative BC (TNBC) vs. luminal BC, and (**g**) downregulated in lobular vs. ductal carcinoma (ca).

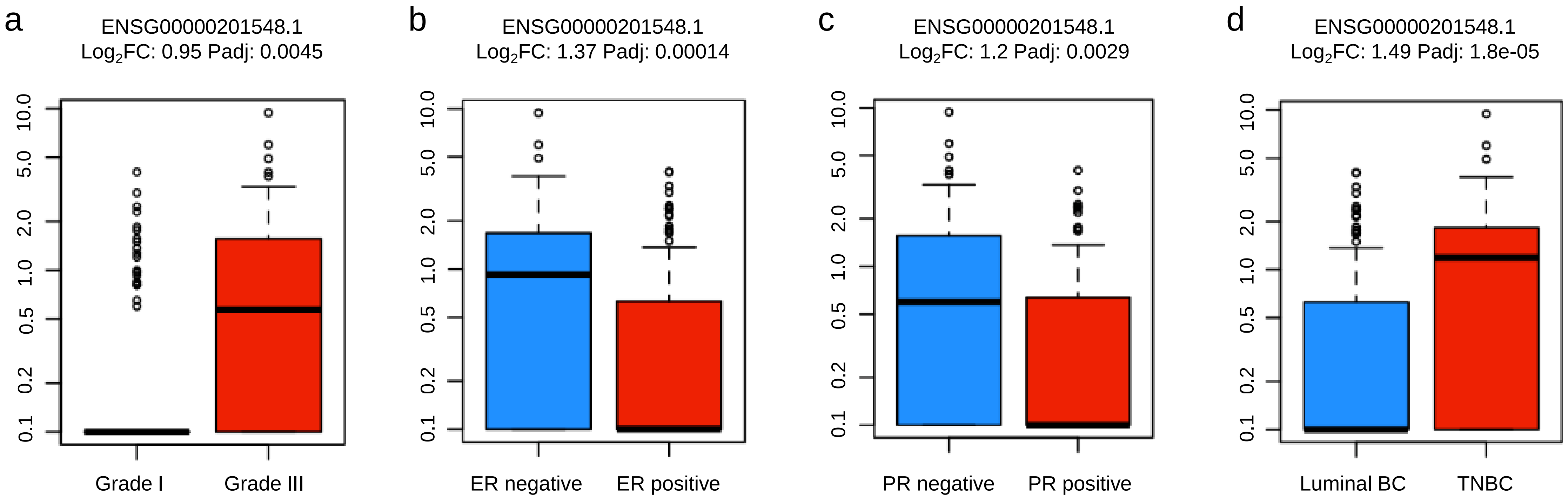

Supplementary Figure S12. The significant associations (*Padj*<0.01) of ENSG00000201548.1 (Y_RNA) with the clinicopathological features of invasive breast cancer (BC). ENSG00000201548.1 was upregulated (**a**) in grade III vs. grade II tumors, (**b**) in estrogen receptor (ER) negative tumors vs. ER positive tumors, (**c**) in progesterone receptor (PR) negative tumors vs. PR positive tumors, and (**d**) in triple-negative BC (TNBC) vs. luminal BC.

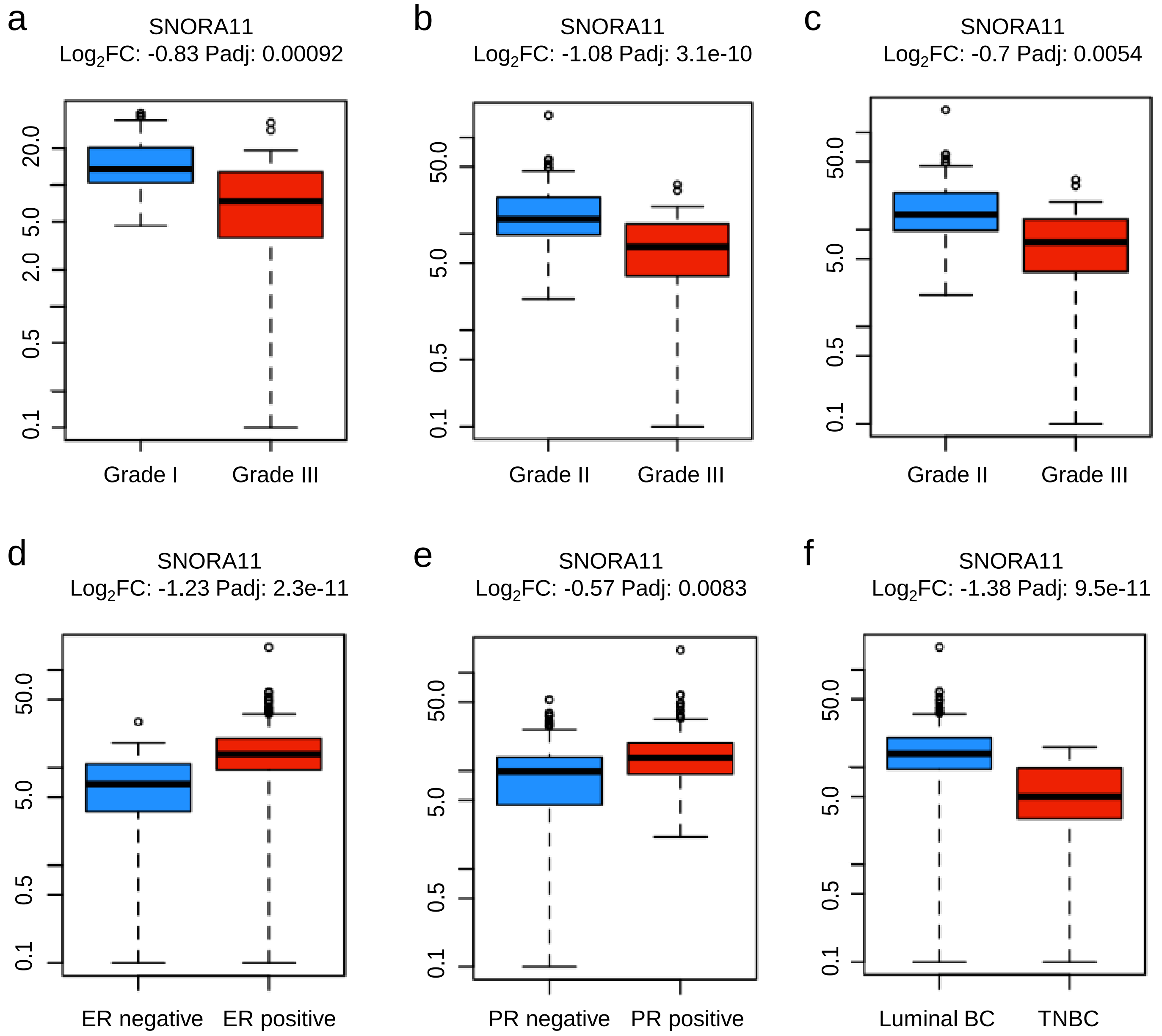

Supplementary Figure S13. The significant associations (*Padj*<0.01) of SNORA11 with the clinicopathological features of invasive breast cancer (BC). SNORA11 was downregulated (**a**) in grade III tumors vs. grade I tumors, (**b**) in grade III vs. grade II tumors, (**c**) in grade III tumors vs. grade II tumors independently of the estrogen receptor (ER) status of the tumors, (**d**) in ER negative tumors vs. ER positive tumors, (**e**) in progesterone receptor (PR) negative tumors vs. PR positive tumors, and (**f**) in triple-negative BC (TNBC) vs. luminal BC.

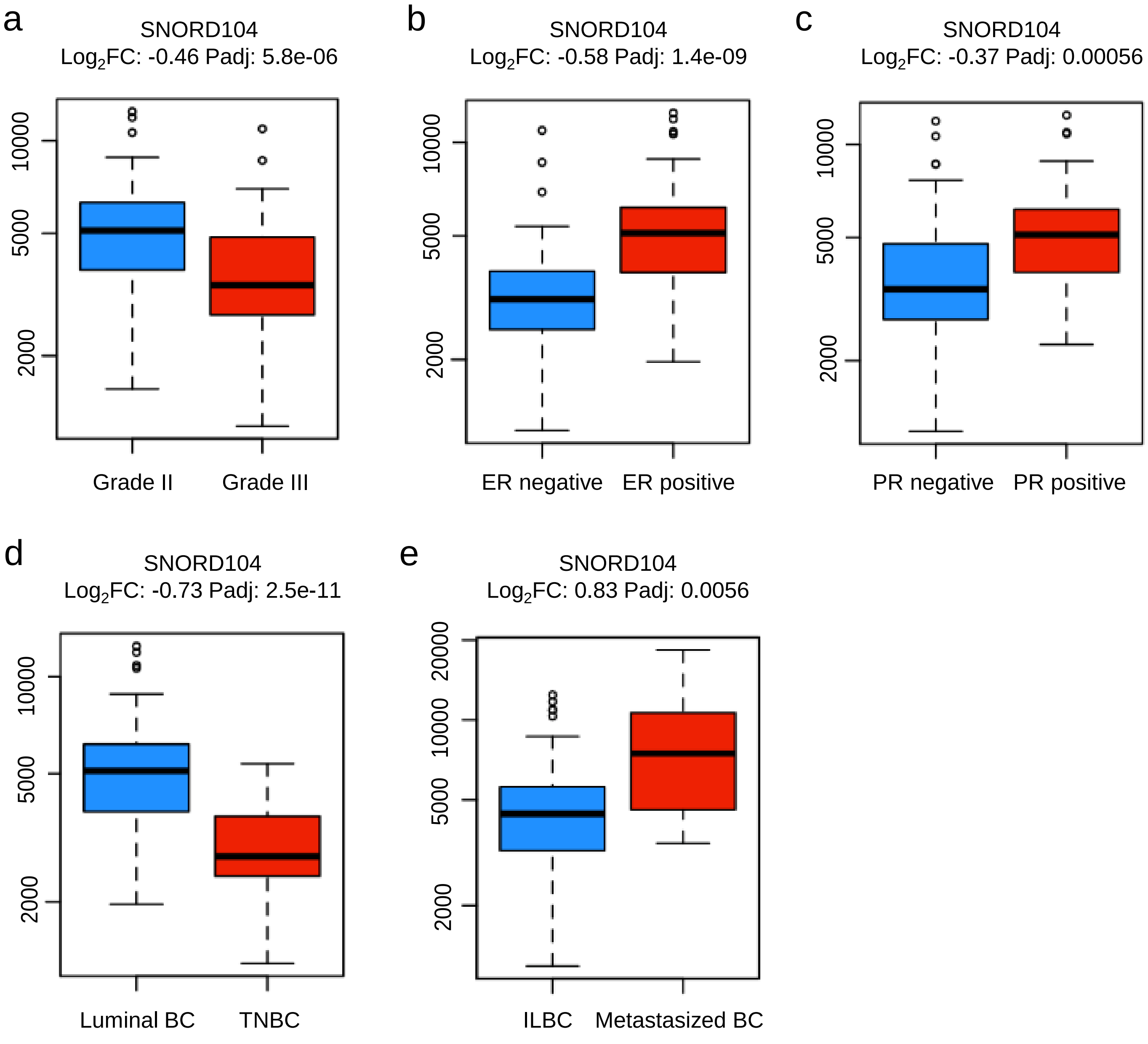

Supplementary Figure S14. The significant associations (*Padj*<0.01) of SNORD104 with the clinicopathological features of invasive breast cancer (BC). SNORD104 was downregulated (**a**) in grade III vs. grade II tumors, (**b**) in estrogen receptor (ER) negative tumors vs. ER positive tumors, (**c**) in progesterone receptor (PR) negative tumors vs. PR positive tumors, and (**d**) in triple-negative BC (TNBC) vs. luminal BC, and (**e**) upregulated in metastasized BC vs. invasive local BC.

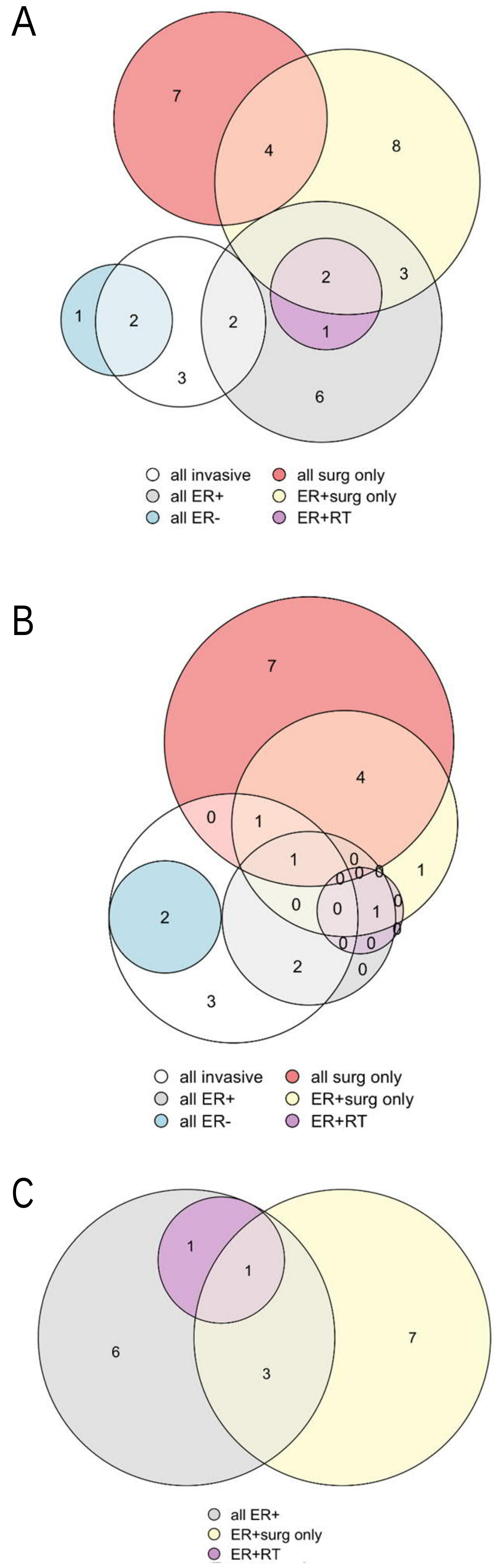

Supplementary Figure S15. Prognostic potential of sncRNAs. Euler diagrams of **A)** the 42 scnRNAs that were identified as possible prognostic markers for invasive local BC, **B)** 23 of which associated with patient outcome independently of tumor ER status, and **C)** 18 that were candidate prognostic markers in ER positive invasive local BC. Note that due to methodological restrictions inherent to multi-group Euler diagrams three sncRNAs are not shown in A) and one in B).

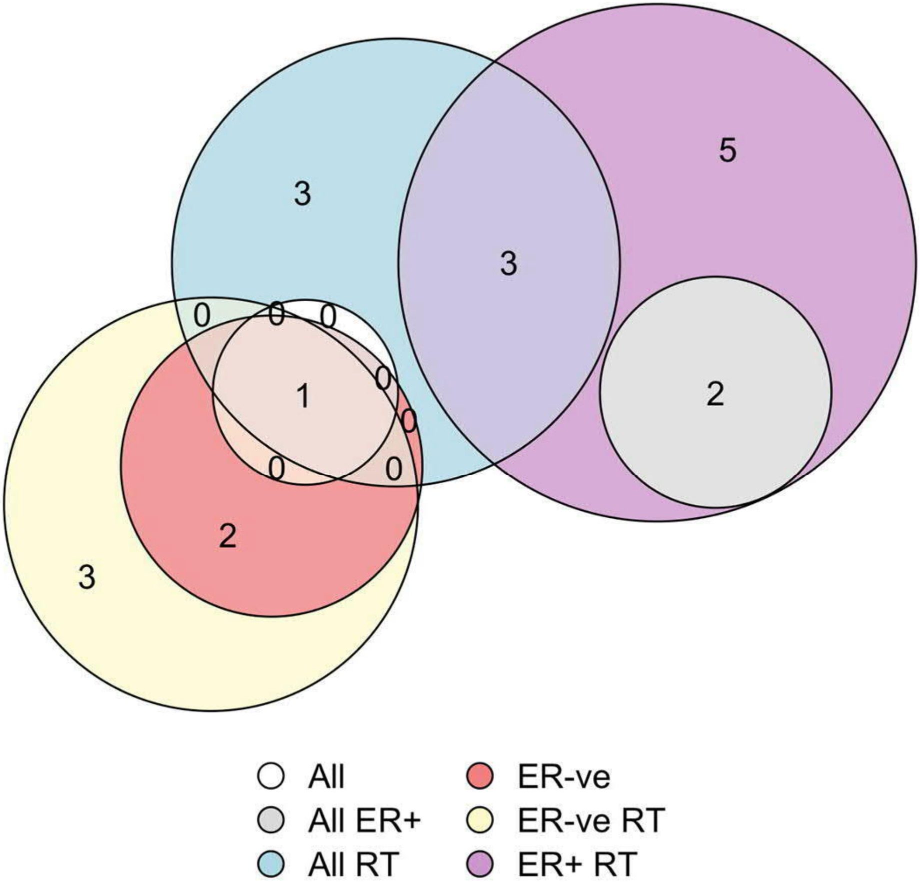

Supplementary Figure S16. Euler diagram of the 20 sncRNAs that were identified as predictive for RT response in the indicated analyses. Note that due to methodological restrictions inherent to multi-group Euler diagrams one sncRNA is not shown. ER-ve, ER negative.
